# Stochastic, Adaptive Sampling of Information by Microvilli in Fly Photoreceptors

**DOI:** 10.1016/j.cub.2012.05.047

**Published:** 2012-08-07

**Authors:** Zhuoyi Song, Marten Postma, Stephen A. Billings, Daniel Coca, Roger C. Hardie, Mikko Juusola

**Affiliations:** 1 Department of Biomedical Science, University of Sheffield, Sheffield S10 2TN, UK; 2 Department of Automatic Control and Systems Engineering, University of Sheffield, Sheffield S1 3JD, UK; 3 Department of Physiology, Development and Neuroscience, University of Cambridge, Cambridge CB2 3DY, UK; 4 Swammerdam Institute for Life Sciences, University of Amsterdam, 1090 GE Amsterdam, The Netherlands; 5 State Key Laboratory of Cognitive Neuroscience and Learning, Beijing Normal University, Beijing 100875, China

## Abstract

**Background:**

In fly photoreceptors, light is focused onto a photosensitive waveguide, the rhabdomere, consisting of tens of thousands of microvilli. Each microvillus is capable of generating elementary responses, quantum bumps, in response to single photons using a stochastically operating phototransduction cascade. Whereas much is known about the cascade reactions, less is known about how the concerted action of the microvilli population encodes light changes into neural information and how the ultrastructure and biochemical machinery of photoreceptors of flies and other insects evolved in relation to the information sampling and processing they perform.

**Results:**

We generated biophysically realistic fly photoreceptor models, which accurately simulate the encoding of visual information. By comparing stochastic simulations with single cell recordings from *Drosophila* photoreceptors, we show how adaptive sampling by 30,000 microvilli captures the temporal structure of natural contrast changes. Following each bump, individual microvilli are rendered briefly (∼100–200 ms) refractory, thereby reducing quantum efficiency with increasing intensity. The refractory period opposes saturation, dynamically and stochastically adjusting availability of microvilli (bump production rate: sample rate), whereas intracellular calcium and voltage adapt bump amplitude and waveform (sample size). These adapting sampling principles result in robust encoding of natural light changes, which both approximates perceptual contrast constancy and enhances novel events under different light conditions, and predict information processing across a range of species with different visual ecologies.

**Conclusions:**

These results clarify why fly photoreceptors are structured the way they are and function as they do, linking sensory information to sensory evolution and revealing benefits of stochasticity for neural information processing.

## Highlights

► Results define how microvilli populations in fly photoreceptors encode information ► Availability and response waveforms of microvilli map light changes to information ► Encoding mechanisms understood through simple adaptive sampling principles ► Sampling principles predict photoreceptor structure and encoding in other species

## Introduction

Fly photoreceptors provide classical model systems for studying how sensory neurons sample and process information. By adapting dynamically to ambient illumination, their voltage responses can represent intensity fluctuations over a truly astronomical scale—from scattered photons of night sky to 10^8^ times brighter daylight [1, 2]—and do so with the fastest temporal resolution known in the animal kingdom. Structural and functional adaptations of fly photoreceptors have been investigated extensively, and we have a wealth of knowledge about their ultrastructure, molecular constituents, and response dynamics [2]. However, we lack a deeper understanding of how these structures and reactions sample and process visual information under different light conditions (but see [3–6]).

To address this, we generated biophysically realistic photoreceptor models and compared their performance to real photoreceptors. In fly photoreceptors, single photon responses (quantum bumps), with variable waveforms and timing (latency distribution), are generated within single microvilli and sum to produce macroscopic voltage responses [7, 8]. By combining stochastic simulations with single cell recordings from *Drosophila* photoreceptors, we show how the number of light-activated microvilli (regarded as elementary sampling units), and the speed and refractoriness of the bumps they generate, dynamically adjust the pool of available microvilli according to the instantaneous photon arrival rate. By extracting the average bump waveforms and latency distributions from real recordings at different light levels and incorporating these into the stochastic models, we quantified how “unreliable” biochemical reactions and refractory sampling units generate reliable neural representations of natural light changes, how increasing the number of sampling units or their sampling speed improve vision, and how feedbacks and stochastic reactions assist encoding. Thus, sensory encoding of naturalistic stimuli can be understood through simple adaptive sampling principles, set by the dynamic availability and variable bump waveforms of the microvilli population. Finally, we demonstrate how these principles can successfully predict the ultrastructure and information transfer of other fly photoreceptors.

## Results

**Figure 1:**
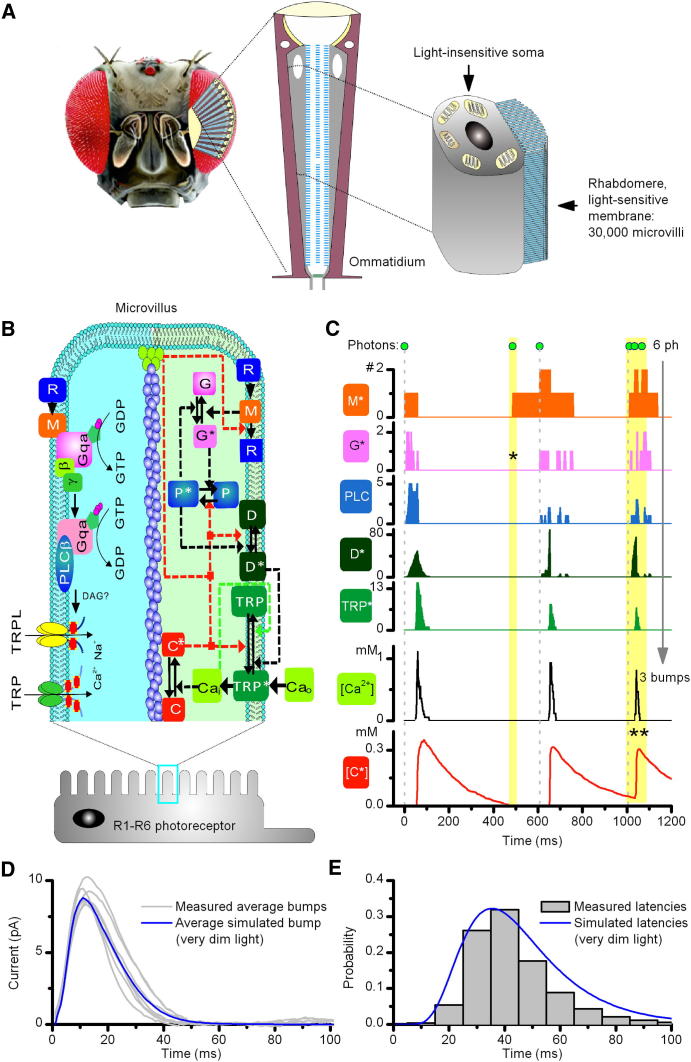
Each Microvillus Is a Stochastically Operating Transduction Unit that Produces Bumps (A) *Drosophila* compound eyes (left) are composed of lens-capped ommatidia (center), each of which contains eight photoreceptors (R1–R8). Right shows schematic of the light-insensitive soma and light-sensitive rhabdomere of an outer photoreceptor (R1–R6). Rhabdomere is made out of 30,000 microvilli. (B) Schematic of phototransduction reactions inside each microvillus. M^∗^, metarhodopsin; C^∗^, Ca^2+^-calmodulin complex, which acts as negative feedback to multiple targets; D^∗^, DAG; P^∗^, G protein-PLC complex. (C) These reactions can be modeled in a stochastic framework, with known molecular interactions, using physiologically measured parameters. Simulated reactions show how a microvillus generates elementary responses (bumps) to captured photons; after a “dead time,” 5-15 TRP-channels open, mediating Ca^2+^ and Na^+^ influx into the microvillus. Ca^2+^-calmodulin complex (red) provides negative feedback, which prevents new bumps until the feedback is low. ^∗^ = G^∗^ activation failed; ^∗∗^ = negative feedback blocked two photon activations. [*C*^⋆^]*_i_* decay phase is longer than the real refractory period, which represents a balance between the feedbacks; the positive feedback can outgrow the negative one in the middle of [*C*^⋆^]*_i_* decay. Thus, a bump can be generated without *C*^⋆^ being zero (e.g., the third bump). (D) Average bumps from seven photoreceptors (whole-cell voltage clamped currents) and an average simulated bump are similar (currents are actually inward: plotted here as outward for consistency). The bump current is computed by equation 11 (Supplemental Experimental Procedures). (E) Latency distributions, including ∼10 ms “dead time,” of simulated and real bumps (data from six wild-type cells) are similar.

To better understand how light information is sampled and processed through the interplay between the phototransduction cascade and voltage-sensitive plasma membrane [8], we constructed a detailed biophysical model of a *Drosophila* outer photoreceptor (R1–R6), based on the dynamics of its molecular components. As in real photoreceptors, the model incorporates two parts (Figure 1A; see Figure S1A and Table S1 available online): a light-sensitive rhabdomere, made out of ∼30,000 microvilli, each of which operates independently as a phototransduction unit, housing an identical stochastic phototransduction cascade (Figures S1B and S1C) and a voltage-sensitive plasma membrane (Figure S1D), which translates the phototransduction current into a voltage response [2, 8].

### Single Microvilli Generate only One Quantum Bump at a Time

We first modeled how a single microvillus (Figure 1B) detects single photons by tracking the numbers and dynamics of photons and cascade intermediates (Figure 1C). Following absorption of one photon by a single rhodopsin molecule, the G protein-based transduction cascade culminates in the opening of 5-15 TRP/TRPL channels within an individual microvillus [9]. The resulting influx of calcium, magnesium, and sodium ions generates a quantum bump [6, 10]. The model shows how dead-time [11, 12] and bump latency distribution [7, 8] arise from the stochasticity, finite speed, and success of these reactions [5, 6]. We see that not all absorbed photons cause bumps; in this particular simulation, six absorbed photons evoked three bumps. Very rarely the molecules downstream fail to activate their targets and the signal fades before the amplification takes off (marked **∗**). In other cases, the reactions are blocked because the photons (marked **∗∗**) arrived during the 100–200 ms “refractory period,” which follows each bump. Critically, calcium provides sequential positive and negative feedbacks (red) to multiple targets, greatly amplifying the signal and accelerating response speed [7, 10]. As soon as a bump is generated, the negative feedback holds the microvillus in a state of inhibition during which it cannot respond to light. This refractory period ends when the negative feedback relaxes and ultimately sets the maximum sample rate (bump production rate) of the microvillus (Figure S2A).

The model further highlights how the bumps adapt to the frequency of the past bumps. Although showing stochastic variation, the first bumps are typically the largest and later ones smaller. Overall, these simulated dynamics generate bumps (Figure 1D) with broadly distributed latencies (Figure 1E) that closely resemble real bumps to very dim illumination, sampled in whole cell patch-clamp recordings of dissociated photoreceptors.

### Microvilli Availability Contributes to Fast Adaptation

**Figure 2:**
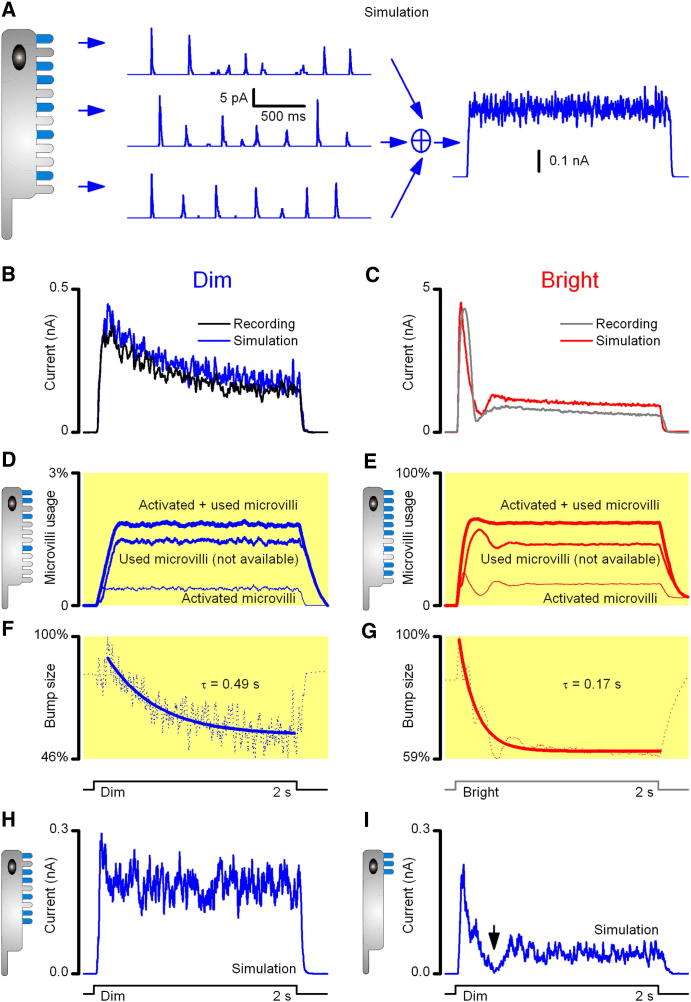
Dynamic Availability of Microvilli Shapes Responses to Light (A) Schematic illustration of the main principle of bump summation. Left side shows that trains of bumps in individual microvilli represent discrete, stochastically generated samples of activation. Right side shows that bumps like these sum to generate a continuous noisy LIC response. (B and C) LICs to dim (3,000 photons/s) and bright (3 × 10^5^ photons/s) pulse stimuli, respectively, recorded from dissociated R1–R6 *Drosophila* photoreceptors during whole-cell patch clamp. Superimposed on these recordings are the simulated LICs to the same dim (blue trace) and bright (red trace) pulse stimuli. LICs are shaped by the number of activated microvilli (shown in D and E) and negative feedback, which reduces the size of the bumps they produce (shown in F and G). (D and E) Output of 30,000 microvilli modeled, showing the fractions of used (refractory) and activated microvilli and their sums in the model simulations to reproduce (B) and (C). Note that microvilli counts are practically immune to changes in negative feedback strength, which reduces their bump size simultaneously (F and G). (F and G) Dotted lines show the difference between normalized LICs (B and C), and the number of activated microvilli (D and E) represents the effect of a reduction in bump waveform on LIC as a function of time (whereas the refractory period affects microvilli usage). During dim stimulation, adaptation (bump size reduction) is slow. In bright stimulation, bump size begins to diminish dramatically already after the first bumps, which shape the initial transient response. Time course of bump adaptation was approximated by single exponentials. (H and I) LICs to a dim pulse stimulus (300 photons/s) of simulated photoreceptors with either 3,000 or 300 identical microvilli. Too few microvilli generate transient responses, because their ongoing photon capture reduces the number of available sampling units for the next round of photons (saturation effect). Therefore, LIC to dim light pulses in photoreceptors with few microvilli (I) looks similar to (but noisier than) the LIC to bright light pulses in photoreceptors with many microvilli (C). In (C) and (I), stochastic (unphased) phototransduction reactions of different microvilli prevent LIC from complete saturation (arrow, no zero DC-signal); even at the start of intense stimulation, there remain microvilli that cannot be light-activated. Refractory microvilli will recover at variable times to the pool of available microvilli (i.e., can then be activated again by light).

An individual microvillus can only produce one bump at a time, and the photoreceptor sums many of them to generate a continuous response, as shown schematically for a dim 2 s step in Figure 2A. With simple summation, such macroscopic responses would be square-like but noisy due to the low bump production rate by microvilli. However, the real light-induced currents (LIC) to light steps, measured under voltage-clamp (Figures 2B and 2C) show additional adaptive trends as the intensity increases. The combined response of few activated microvilli to a dim stimulus is smaller, slower, and noisier (Figure 2B) than that of many activated microvilli (Figure 2C) to a bright stimulus, which reaches a transient peak that rapidly decays and rebounds, before gradually settling to a steady-state (plateau). Here, fast adaptation happens during the early transient response (Figure 2C), whereas slow adaptation establishes the slower trend toward the plateau (Figure 2B).

To elucidate the processes behind fast and slow adaptation, we fixed all free parameters in our model and simulated the output of 30,000 microvilli to dim (3,000 photons/s) and bright (3 × 10^5^ photons/s) pulses. We first assume that each of the microvilli operates independently and the macroscopic LIC response is a summation of the bumps they produce [2, 7].

The results imply that fast adaptation, i.e., processes shaping the early transient response, depends directly on the number of microvilli that can participate in the response, determined jointly by the intensity and (stochastic) duration of the refractory period. During dim stimulation at low photon rates (Figure 2B), only a small fraction of the microvilli is activated (or refractory) at any one time (Figure 2D), leaving a large pool of unused microvilli to sample further incident photons. By contrast, at the onset of a bright stimulus (Figure 2C), a large fraction of the microvilli is activated simultaneously but then becomes refractory (Figure 2E). This leaves only a small fraction of the microvilli to respond to the next photons in the stimulus until more microvilli become available again (see also [13]). Therefore, the number of activated microvilli shows a large initial peak, followed by a rapid drop that then settles to a steady-state as photon arrivals and refractory periods become decorrelated. Thus, the early transient response (Figure 2C) is evoked largely by the initial bumps from a large pool of microvilli (Figure S2B) and its width is largely shaped by bump latencies (Figure S2C), which vary in dark and light adaptation and with temperature [7, 8, 14].

If all bumps were identical, then the macroscopic current would simply represent the number of activated microvilli at a given photon rate, with a flattened steady-state response (Figures 2D and 2E). However, compared to these microvilli counts, the measured light-induced currents to dim light show a slowly decaying trend (Figures 2B and 2D), whereas the plateau levels in response to bright light are much smaller (Figures 2C and 2E). This is because bumps adapt, becoming smaller and briefer with brighter backgrounds [8, 15, 16]. These effects have been modeled in the simulated light currents to dim and bright pulses by introducing a global negative feedback parameter, *n_s_*, which increases exponentially with time and accelerates with increasing intensity (Table S2). This global feedback presumably represents Ca^2+^-dependent inhibition or adaptation due to light-induced calcium spread between microvilli via the cell body [2]. This attenuates bump waveforms (Figures 2F and 2G) by progressively strengthening the negative feedback to multiple targets [2, 8, 15, 17]. Thus, reduction both in the number of activated microvilli [13, 18] and in their bump waveforms largely accounts for the transient dynamics of the LIC response to bright stimuli, whereas calcium-induced bump adaptation accounts for key aspects of slow adaptation including the reduced plateau response.

Whereas Ca^2+^-dependent light adaptation is well established [2, 17, 19], our analysis indicates for the first time how sampling by microvilli leads inevitably to adaptation by stochastic saturation of transduction units. To make this structural connection more obvious, we consider simulations, in which a dim light input (300 photons/s) is sampled either by 3,000 or 300 microvilli. For the given photon rate, 3,000 microvilli can generate a macroscopic response (Figure 2H) that is comparable to a real LIC (Figure 2B), but with only 300 microvilli, the summed response shows early saturation (Figure 2I). However, crucially, the responses always recover to a steady-state output level, as set by the dynamic ratio of used and available microvilli, but this output level is higher in a photoreceptor with more microvilli. Thus, although the simulations confirm two classic hypotheses: that the microvilli represent transduction units [20] and that the number of transduction units determines the maximum rate at which photons can be sampled [21], they also reveal new insight into light adaptation and the design of microvillar photoreceptors.

### Global Feedbacks Affect All Microvilli

**Figure 3:**
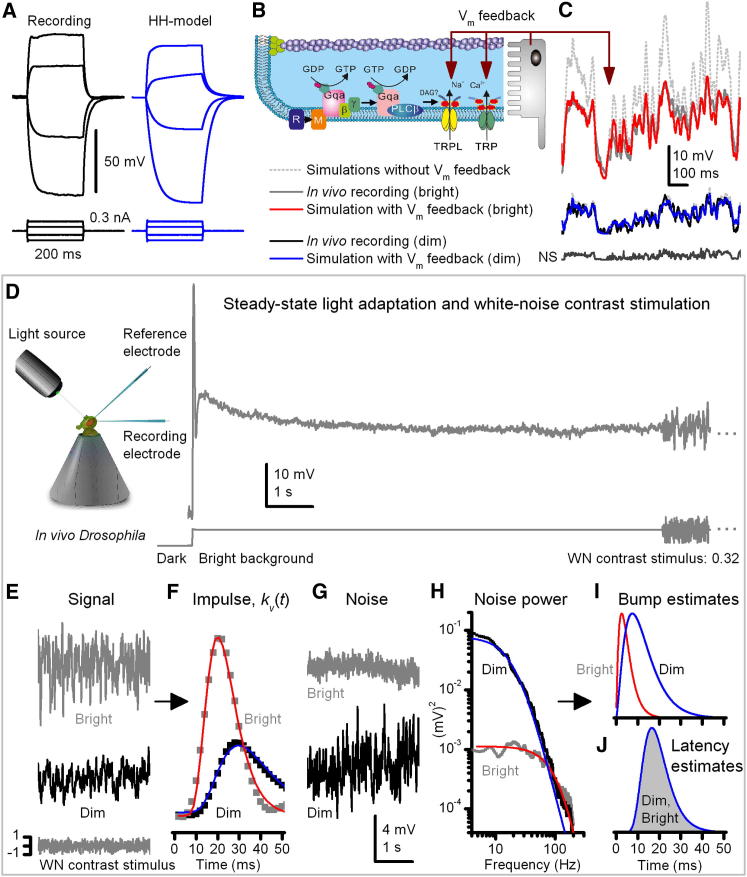
Stochastic Microvilli Model with a Hodgkin-Huxley Type Membrane Model Generates Realistic Voltage Responses, Providing a New Framework to Dissect How Photoreceptors Sample Changing Light Information (A) In vivo intracellular (left) and simulated (right) voltage responses to current pulses in darkness. (B) Depolarization causes a global negative voltage feedback, which reduces the driving force for cations entering microvilli through TRP/TRPL channels. (C) Voltage feedback compresses bumps and macroscopic responses, scaling the simulated responses realistically. With voltage feedback, simulated responses to naturalistic light intensity series (dim and bright) mimic the real recordings. Models without voltage feedback used light-adapted bumps but had light-adapted membrane resistance and dark resting potential; their macroscopic responses are shown with gray dotted lines. (D) In vivo experiments; light background adapts a photoreceptor to a steady-state, where its voltage responses to white-noise (WN) contrast stimulus can be analyzed to obtain the average bump waveform and latency distribution [8]. (E) Average responses (signals) to repeated WN contrast at dim (∼1,500 photons/s) and bright (∼1.5 × 10^5^ photons/s) illuminations (backgrounds). (F) Linear impulse responses (squares) approximate how photoreceptors convert dim and bright WN stimuli to voltage responses; impulse responses are fitted with log-normal functions (red and blue traces) [42]. (G) Noise, the difference between signals and responses, is predominantly bump shot noise [8]. (H) Corresponding noise power spectra. (I) Average bump estimates were analytically converted from noise power spectra's gamma-distribution fits (H; smooth traces) [8, 15]. (J) Bump latency distributions for dim and bright illuminations, obtained by deconvoluting average bump estimates from the fitted impulses [8], are virtually identical (shown here by a single blue trace; gray interior indicates distribution). The stochastic photoreceptor model can then be set to operate with these bump statistics (cf. Figure 4).

Thus far, we have considered only the light-sensitive conductance under voltage-clamp conditions. Next, we extended the modeling to the voltage domain by allowing interplay between light- and voltage-gated ion channels and compared our results with intracellular voltage recordings with sharp microelectrodes. We used a detailed Hodgkin-Huxley type model of a typical *Drosophila* photoreceptor's plasma-membrane, which incorporates a suite of voltage-sensitive K^+^-channels and accurately simulates voltage responses to current pulses [22, 23] (Figure 3A). Here, we further allow the voltage of the plasma membrane to feed back to the phototransduction cascade by regulating the electromotive force for the light-sensitive current across all microvilli (Figure 3B; Figure S3A), as must happen in vivo. As the photoreceptor depolarizes to a light stimulus, both the battery that drives calcium and sodium ions through TRP channels and the membrane resistance reduces throughout the cell, adapting (attenuating) bumps in every active microvillus, irrespective of their bump history. Thus, together intracellular voltage and calcium accumulation (Figures 2F and 2G) provide global feedbacks, which affect all microvilli, causing a divisive nonlinearity [24] that dynamically adapts their bump sizes to the ongoing photon rate, as supported by noise analysis [8, 14, 15, 25]. Consequently, the macroscopic voltage response becomes compressed (Figure 3C). Furthermore, the transition from prolonged dark adaptation to light adaptation [7, 8], which narrows the width of the early transient voltage responses, can be replicated by narrowing the bump latency distribution (Figures S2C–S2E) and understood as a memory of light exposure [26], presumably mediated by intracellular calcium accumulation affecting the microvilli globally.

### Microvillar Bump Production Dynamics Set Information Rate

To examine how *Drosophila* photoreceptors sample and process visual information in different light-adapted states, we recorded voltage responses to repeated pseudorandom (white noise) and naturalistic (collected from natural environment [1]) contrast sequences at six different intensity levels in vivo at 25°C. By using established noise analysis techniques on the responses to the white noise sequence [8, 12, 14, 26], we extracted the average bump shapes and their latency distributions under different adaptational states (Figures 3D–3J). As previously reported, the average bumps were larger and slower at dim intensities and smaller and faster at brighter illumination (Figure 3I), whereas the bump latency distributions, in even a weakly light-adapted state (Figure 3J), were narrower than following prolonged dark adaptation (Figure 1E), but then changed little during adaptation to further increasing background intensity [8].

**Figure 4:**
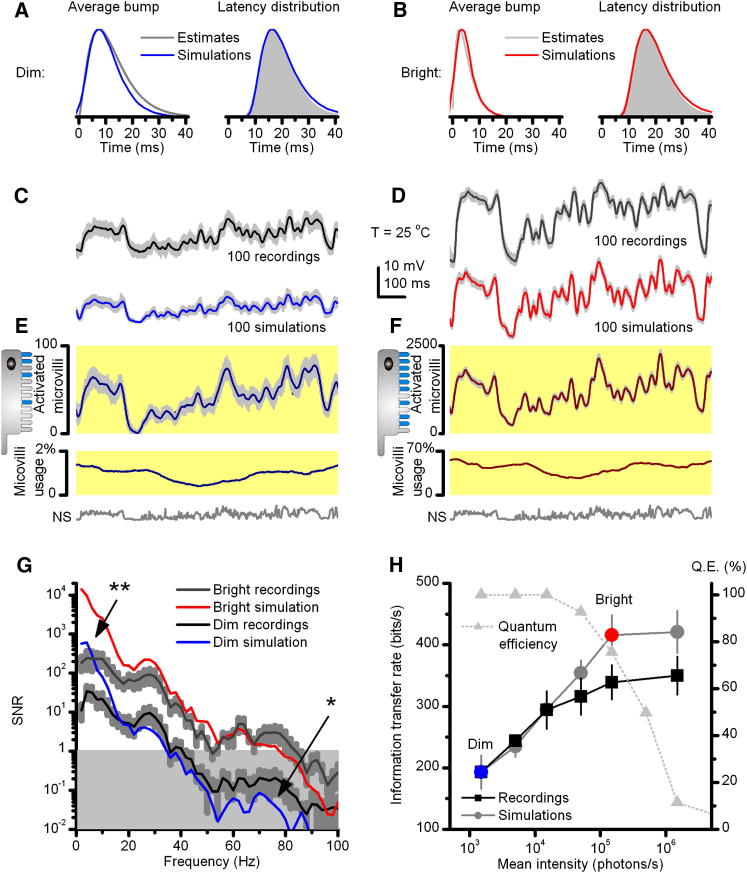
Encoding Performance of Real and Modeled *Drosophila* Photoreceptors to Naturalistic Stimuli (NS) Match, Showing that Adaptive Sampling Increases the Flow of Information (A and B) In the simulations, stochastic microvilli models were set to use the corresponding average bump waveforms and latency distributions (here normalized for clarity), estimated from the white-noise contrast experiments at different illuminations (dim and bright; Figures 3H–3I). This was done by refixing two model parameters: *n_s_* for the bump shape and *la* for the latency distribution (see Supplemental Experimental Procedures). (C and D) One hundred superimposed in vivo responses (light gray) and their average signals to repeated naturalistic dim and bright stimuli (equal contrast), respectively, and the corresponding simulations. (E and F) NS activate microvilli stochastically with appropriate dynamics and statistics for the given mean illumination (A and B). Due to low-passing input (1/f-statistics; [1]), the number of activated microvilli is mostly responsible for the corresponding response waveforms (above). This encoding uses only a fraction of 30,000 microvilli (in repeated NS, maximally ∼68%) because NS contain long relatively dim periods, allowing refractory microvilli to return to the pool of available ones during stimulation. (G) The corresponding signal-to-noise ratios (SNR) of real (mean ± SD, n = 5) and simulated responses. ^∗∗^ marks the difference at lower frequencies, probably due to slow adaptation, instrumental noise and damage, which the model lacks; ^∗^ marks extra information at higher frequencies, probably due to input from other cells in the network, which the model lacks. The overall shape of SNRs reflects 1/*f* statistics of NS, as dominated by its low-frequency content. (H) Mean rate of information transfer of two best photoreceptors (black) and the model (gray) to the same NS at six different illuminations. Recordings' lower information transfer at three brightest intensities can be attributed to damage, experimental noise, and intracellular pupil, which progressively filter out photons. At dim intensities, the effect of experimental noise and adaptation may be compensated by the synaptic network introducing new information from neighboring photoreceptors [34, 40]. Grey dotted line shows how the quantum efficiency (Q.E.) of simulated photoreceptor output drops with brightening NS, while the information transfer approaches a constant rate. Error bars show SD.

The estimated bump shape and latency distribution statistics at each light intensity level were incorporated into the full model by refixing two negative feedback parameters: *n_s_* and *la* (Figures S3B and S3F), which regulate the strength and speed of the Ca^2+^-dependent negative feedback in each simulated microvillus (Figures 4A and 4B). By simulating bump production over all 30,000 microvilli, the model was then used to predict microvilli availability and macroscopic voltage responses during repeated naturalistic stimulation (NS) at each light intensity level.

Because the model's bump statistics were now fixed to the steady-state adapted natural bump variations at any one background, the close correspondence of experiment and simulation indicated that voltage responses of fully light-adapted photoreceptors to naturalistic stimuli (Figures 4C and 4D) can be largely accounted for by the changing fraction of their activated microvilli (Figures 4E and 4F). With dim stimulation (low photon rate), this sampling utilized <2% of microvilli, allowing detection of essentially all absorbed photons. With bright stimulation, maximal microvillar usage approached 62% (mean usage: 48%). With increasing background intensities, the increased probability of photons arriving during a refractory period resulted in steep fall-off in quantum efficiency but with no loss of information transfer (Figure 4H). The encoding was logarithmic; larger bumps to dim NS generated macroscopic responses, which reached ∼1/3 of those to bright NS, although the dim and bright intensities differed by 100-fold. Such gain control is due to a combination of passive and active membrane and reduction in bump current, which is reduced to <1 pA during bright stimulation. Crucially, the responses predicted by the model, their signal-to-noise ratios (SNR; Figure 4G), and rate of information transfer (Figure 4H), as quantified from their capacity to distinguish different stimulus values [27], closely resembled those of actual recordings at each light level. Minor discrepancies between model and experiment can be readily accounted for by factors not included in the model. Thus, under bright stimulation, the simulations had higher information content due to their higher low-frequency SNR (Figure 4G, marked ^∗^). This difference is probably because the model lacks an intracellular pupil, which progressively filters out photons [21, 28], and additional slow adaptation mechanisms, such as translocation of components of the transduction cascade [29–32]. These processes, together with any damage, recording, or instrumental noise, increase variability in low frequency responses of real cells. The model also lacks regulation from synaptic feedbacks [33, 34], which spread extra information from other photoreceptors in the network, boosting high-frequency signals (SNR) [34–36] (Figure 4G, marked ^∗∗^). Nonetheless, these results indicate that the photoreceptor output mostly represents a complex function between the biophysical sampling rate (bump production rate) and sample size (bump waveform).

### Benefits of Stochasticity and Microvillar Feedbacks

If microvilli generated identical bumps with everything else being equal, then for the same slowly changing light input, two macroscopic outputs, one integrated from small and the other from large bumps, have the same rate of information transfer (simulated LICs: Figures S4A–S4D). This is because bump shape affects signal and noise equally and can be deconvolved, as a known filter, from the outputs, revealing their identical inputs (data processing theorem [27, 37]). A photoreceptor's rate of information transfer, therefore, depends fundamentally on changes in its bump production rate. However, when real photoreceptors adapt to a certain light level, the feedback mechanisms within microvilli make encoding more than just a summation of similar or independent bumps. Bumps generated in individual microvilli have adapted (albeit stochastic) bump waveform and refractory periods that reflect their specific absorption histories and which affect information in the macroscopic responses.

**Figure 5:**
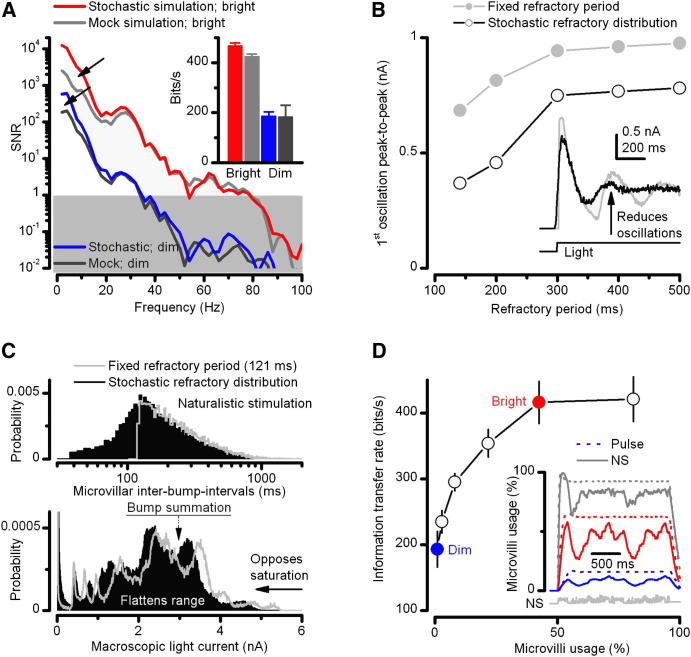
Benefits of Stochasticity and Microvillar Feedbacks on Sampling Light Information (A) Adapted variability of bump waveforms increases low-frequency SNR (arrows) in the macroscopic light current responses to dim and bright naturalistic stimulation. Here, stochastic simulations are compared to mock simulations, in which responses were integrated from decorrelated bumps, taken randomly from the same stochastic bump amplitude and duration distributions. Hence, bump variations even at steady-state adapted conditions are not random but correlate partly with the light history (through the feedback mechanisms within microvilli). These improvements in low-frequency SNR, though, have only a small impact on the rate of information transfer (inset). Responses and their variability are compared in Figures S5H and S5I, respectively. (B) Light currents, integrated from bumps with stochastic refractory distributions, oscillate less than those integrated from bumps with fixed refractory periods. Here, tested for the first oscillation after the initial transient. (C) Stochastic refractory period distribution utilizes the available microvilli more evenly than a fixed refractory period, as seen by its broader inter-bump-interval probability distribution (above). Bumps with stochastic refractory periods integrate macroscopic responses that utilize less (opposing saturation), but more evenly, a photoreceptor's output range (below). In optimal sampling, every microvilli is used equally often. Inter-bump-interval distributions were calculated for 30,000 microvilli individually and the probability over the whole population. Multiple peaks in the probability distribution (below) are characteristic for voltage responses to natural light intensity time series stimuli [1]. (D) A photoreceptor's rate of information transfer reaches maximum when about 50% of its microvilli are in continuous use and is maintained, despite steep fall-off in quantum efficiency (QE) at the brightest intensities (Figure 4H). Inset shows that during very bright naturalistic stimulation, because of its 1/*f* statistics that contain interspersed darker periods (negative contrasts), proportionally more microvilli recover than during light pulses (thin dotted lines) of equal mean intensity. A very bright pulse (10^6^ photon/s) activates ∼99% of microvilli at the beginning of stimulation, and their usage remains high throughout the pulse. Error bars show SD. (A and D) Bright and dim indicate 3,000 and 3 × 10^5^ photons/s.

Interestingly, simulations indicate that bumps with such adaptive correlations generate less low-frequency noise during response summation than a mock series of bumps with the same distributions but lacking such correlations (Figure 5A). In addition, a stochastic refractory period distribution suppresses oscillations in macroscopic responses (Figure 5B) and opposes saturation by using microvilli and photoreceptor output range more evenly (Figure 5C). But most importantly, although the equilibrium between the used and available microvilli ultimately limits the bump production rate at very bright stimulation of high SNR [38] (Figure 5D), failures in bump production, whether stochastic or due to refractory period, promote output constancy. As long as the photoreceptor maintains its maximal bump production rate, its output will have the same rate of information transfer, irrespective of whether its microvilli were bombarded by 10^5^ or 10^6^ photons/s [8, 34, 39]. Furthermore, because microvilli will stochastically flip between processing states, even in very intense light, some will always return to the active “available pool,” making it difficult to inactivate all of them at once. Thus, the models show how microvillar feedbacks and stochasticity of sampling resist saturation and enable more invariable neural information capture.

### Information Transfer in Different Fly Photoreceptors Is Also Predictable

**Figure 6:**
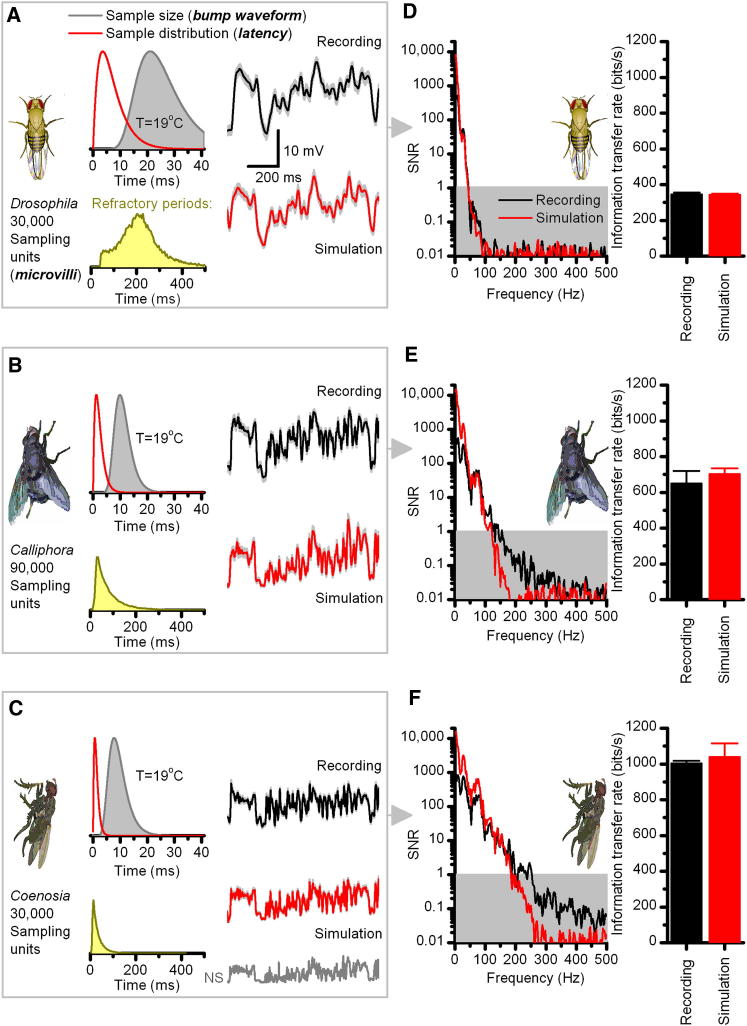
Photoreceptor Models of Adaptive Sampling Behave Like Real Photoreceptors (A–C) Photoreceptor output of *Drosophila*, *Calliphora*, and *Coenosia*, respectively, were simulated by stochastic models. We fixed the number of microvilli and approximated their average bump waveforms (dark red) and latency distributions (gray) from in vivo recordings by adjusting the negative feedback strength within their microvilli (by refixing two global negative feedback parameters: *n_s_* and *la*; details in Supplemental Information). These photoreceptor models' voltage responses (red) to the repeated presentations of a naturalistic stimulus (NS) pattern behave as their real counterparts (black). Lower insets show the corresponding refractory periods (inter-bump-intervals, yellow), generated by the microvilli of the models to NS. In (A), because of the lower temperature (19°C), this distribution is wider than the one in Figure 5C (25°C). (D–F) Respective signal-to-noise ratios (SNR) and the corresponding information transfer rates of the simulated responses follow those of the real recordings.

Lastly, we extended this framework to sensory evolution by building stochastic models of R1–R6 photoreceptors in three different fly species (Figure 6): *Drosophila*, blowfly (*Calliphora vicina*), and killer fly (*Coenosia attenuata*), based on intracellular recordings at 19°C. Each of these flies has a different lifestyle (predatory or herbivorous) living in specific habitats, from shady forests to sunny shores [39]. Presumably to accommodate such differing visual demands, their rhabdomeres have evolved appropriately, hosting different numbers of microvilli with different reaction speeds [39]. Again, through noise and signal analysis (cf. Figure 3D), we extracted the average bump waveforms and latency distributions from their voltage responses to a pseudorandom contrast stimulus in vivo, and these statistics were incorporated accordingly in each model (Figures 6A–6C). Moreover, the properties of their voltage-sensitive membranes were approximated with Hodgkin-Huxley type membrane models, with parameters adjusted to simulate in vivo recordings to current injections (Figure S5).

In each case, simulated responses (red) closely resembled the real recordings (black) to the repeated naturalistic stimulus. Here, slow-flying *Drosophila* has the fewest microvilli (Figure 6A), with the slowest phototransduction reactions (see [39]), generating the widest latency distribution, slowest bumps, and longest refractory periods. Such low-passing sampling limits the SNR of responses at high frequencies, communicating 350 bits/s at daylight intensities, >10^5^ photon/s (Figure 6D). Interestingly, this information transfer rate approximates that for the same stimulus at a higher temperature of 25°C (Figure 4F), suggesting that insect photoreceptors may have evolved to provide consistent representations of naturalistic image statistics in varying environmental conditions, in agreement with earlier suggestions [26]. In contrast, the fast-flying scavenger, *Calliphora*, has three times more microvilli (Figure 6B), which operate three times faster, extending the range of reliable signaling to three times higher frequencies and so doubling the rate of information transfer (Figure 6E). The small *Coenosia* has a similar number of microvilli to *Drosophila* (Figure 6C), but its phototransduction is by far the fastest of these species, enabling the largest sample rate changes. Its fast bumps, brief latencies, and short refractory periods generate the highest high-frequency SNR and rate of information transfer (Figure 6F), as required by its demanding predatory lifestyle of fast aerobatic hunting [39].

At high stimulus frequencies, the SNR of the *Calliphora* and *Coenosia* recordings exceeds that of the simulations (Figures 6E and 6F). Because later transformations by membrane filtering cannot improve their encoding performance, affecting signal and noise equally [27, 37], this improvement probably comes through functional contacts, which channel in new information from other photoreceptors [34] and stored information from the network [36, 40] in activity-dependent manner.

## Discussion

Using experiments and modeling, we have shown how stochastic adapting sampling principles, which can be directly derived from biochemical and biophysical reactions occurring within a finite population of microvilli, govern information processing in fly photoreceptors.

### Adaptive Sampling Promotes Contrast Constancy while Enhancing Novel Events

Our results suggest that by compartmentalizing its inherently stochastic biochemical reactions into semiautonomous transduction units (microvilli), phototransduction in flies evolved to generate reliable neural representations of their visual environment. Adaptive sampling by microvilli relies upon local stochastic reactions and global nonlinear interactions (calcium and voltage feedbacks) to encode natural contrast changes. By adjusting the microvilli availability and the speed and the size of their individual bumps, local stochasticity and global feedbacks promote matching of the information transfer rate of the macroscopic voltage responses with the ecological demands of each fly species, approximating contrast constancy under varying conditions.

**Figure 7:**
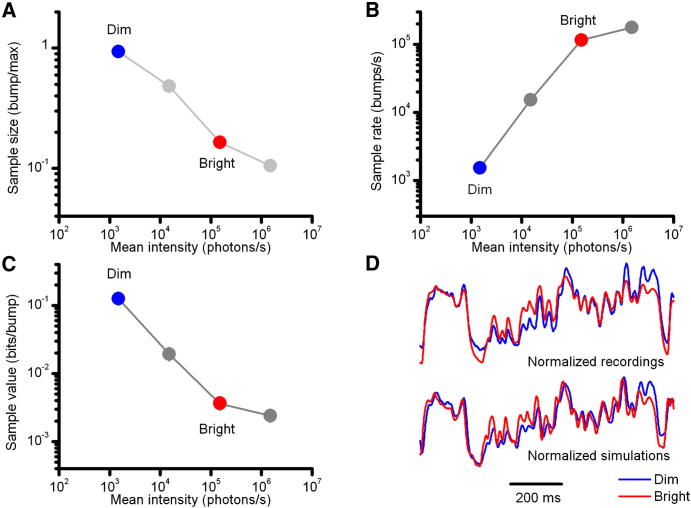
Adaptive Sampling Promotes Contrast Constancy With brightening naturalistic stimulation: (A) The sample size (bump amplitude) is attenuated. (B) More microvilli are activated, increasing sample rate, until progressive reduction in quantum efficiency stabilizes a photoreceptor's bump output. (C) These dynamics (A and B) reduce the amount of information each sample (bump) carries (D) but ensure that relative changes in voltage responses represent naturalistic light changes (contrasts) accurately, irrespective of the ambient illumination. Normalized voltage signals (n = 100 repetitions) to the same naturalistic contrast stimulus shown for both real and simulated *Drosophila* photoreceptors at dim (1,500 photons/s) and bright (1.5 × 10^5^ photons/s) illuminations. Although contrast gain in absolute terms (voltage/unit contrast) increases with light intensity [8, 12, 26, 34, 43], the temporal structure of the transmitted signal remains practically invariable.

In dim conditions, every photon counts, and the quantum efficiency of *Drosophila* photoreceptors is probably close to 100%. The resulting bumps are at their slowest and largest (Figure 7A) [8] and are integrated to generate consistent voltage responses to slow contrast changes, which dominate in the 1/f statistics of the natural scenes. In bright conditions, however, many microvilli are refractory (not available), reducing quantum efficiency. This desensitization stabilizes the bump production rate below saturation (Figure 7B) and, with the help of the global feedbacks, tends to equalize the allocation of visual information within the photoreceptor's limited signaling range (Figures 3C and 5D). Individual photons carry now less value (Figure 7C), and the resulting bumps are small and fast [8], integrating to generate robust macroscopic voltage responses; but again the responses dominantly represent slower contrast changes, attributable to the 1/f statistics. Importantly, the adaptive stochastic bump shape and refractory period distributions smoothen integrated responses (improving low-frequency SNR; Figure 5A) and resist oscillations to sudden, large light changes (Figure 5C). For these reasons, the macroscopic (normalized) voltage responses to light contrasts are highly similar at different illuminations (Figure 7D) [see also: 26], capturing the temporal pattern in the image sequences, as projected by the lens system of a fly in motion [1].

Adaptive sampling also provides an intrinsic capacity to enhance novel events. Although after phototransduction subsequent adaptation cannot increase the photoreceptor's rate of information transfer [27], during sampling it can if it produces a differential change of signal relative to noise between successive responses to the same stimulus. Simulations indicated that the first “towering” response to a bright step contains more samples, and thus has a higher signal-to-noise ratio than subsequent responses, for which less microvilli are activated. Similarly, the first, negative voltage response to a dim step will be enhanced because more microvilli will be in an inactive state than in subsequent responses, because initially many are still refractory [41]. Accordingly, photoreceptors' information transfer is higher at large dim-to-bright or bright-to-dim stimulus transitions and decreases afterwards in correlation with the adaptation to the stimulus [27, 34, 36]. Thus, not only does adaptive sampling lead to robust encoding of natural light changes over the full dynamic range of environmental light intensities, it also enhances novel or surprising stimuli, which generate the largest sample rate changes (increments or decrements) in respect to the ongoing average.

## Supplemental Information

Document S1. Figures S1–S5, Tables S1 and S2, and Supplemental Experimental Procedures
