# Supplementary material for: Stochastic, Adaptive Sampling of Information by Microvilli in Fly Photoreceptors
Source: Curr Biol. 2012 Aug 7;22(15):1371–80. doi: 10.1016/j.cub.2012.05.047 (PMC3420010; doi:10.1016/j.cub.2012.05.047)
Supplement: Document S1. Figures S1–S5, Tables S1 and S2, and Supplemental Experimental Procedures [file mmc1.pdf]

Current Biology, Volume *22*

Supplemental Information

Stochastic, Adaptive Sampling of Information by Microvilli in Fly Photoreceptors

Zhuoyi Song, Marten Postma, Stephen A. Billings, Daniel Coca, Roger C. Hardie, and Mikko Juusola

Supplemental Inventory

1. Supplemental Figures and Tables

Figure S1, related to Figure 1

Figure S2, related to Figures 1–3

Figure S3, related to Figure 3

Figure S4, related to Figure 5

Figure S5, related to Figure 6

Table S1, related to Figures 1–7

Table S2, related to Figure 2

2. Supplemental Experimental Procedures

3. Supplemental References

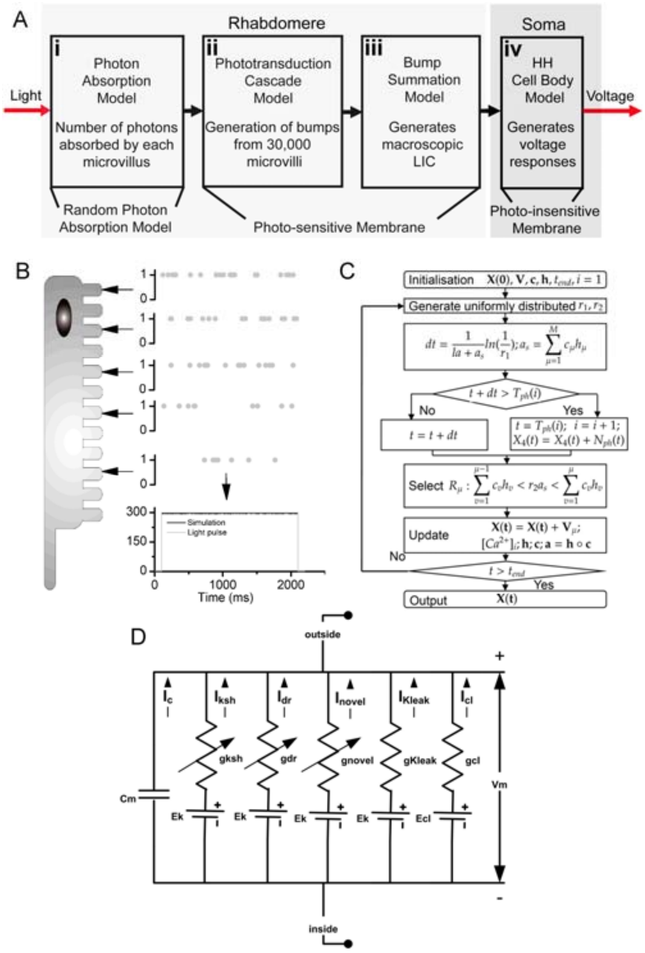

Figure S1. Schematic *Drosophila* Photoreceptor Model Structure and Definitions for Bump Parameters, Related to Figure 1

(A) *Drosophila* photoreceptor model consists of four modules: i. Random photon absorption model. ii. Model for the phototransduction cascade in a single microvillus. iii. Integration of LIC from 30,000 microvilli. iv. Hodgkin-Huxley model for the photoreceptor cell membrane [1, 2].

(B) Modeling photon absorption in microvilli. Light pulse contains $3x10^5$ photons/s, which are absorbed by a population of microvilli according to Poisson statistics. Each microvillus absorbs photons discontinuously, generating a photon absorption series (gray dots). Only very rarely one of them may absorb 2 photons within the same time interval. 1 ms time interval was used.

(C) Flow chart of the stochastic bump model for photon series input. X is the state vector. V is the state transfer matrix, which is composed of M vectors: $\mathbf{V} = [V_1, V_2, \ldots, V_M]$. In the reaction R_μ, the state is updated by V_μ. h is the M-length vector of reactant-pair numbers. c is the M-length vector of stochastic reaction-rates. a is reaction-propensity vector and '$\circ$' in the operation of '$a{=}h{\circ}c$' means element wise product. t_{end} is the termination simulation time, T_{ph} is a vector recording the time point when there is photon stimuli, and i is iteration index for T_{ph}.

(D) Electrical circuit of the HH-cell membrane model of *Drosophila* R1-R6 photoreceptor. Cell membrane is modeled as a capacitor, voltage-gated channels as voltage-regulated conductances, leak channels as fixed conductances, reversal potentials for different ion species as DC-batteries. Abbreviations: ksh: Shaker; dr, delayed rectifier; novel, novel K^+; Kleak: K^+ leak; cl, chloride leak conductance.

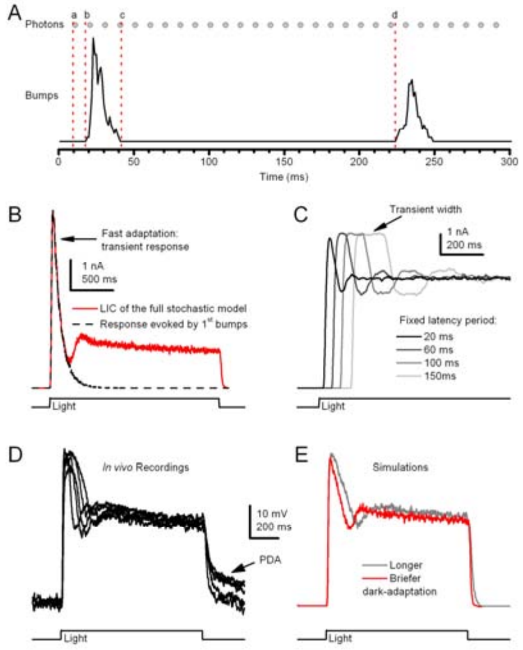

Figure S2. Definition of Bump Parameters and Characterization of How Latency Affects Fast Adaptation, Related to Figures 1–3

(A) Bump latency is the time interval from photon stimulus to the first opening of TRP/TRPL channel (a to b). Bump duration is the duration from the first opened TRP/TRPL channel to the last closed TRP/TRPL channel of a bump (b to c). Refractory period is the time interval between two bumps minus the latency for the second bump. The exact latency for the 2nd bump during continuous photon stimuli is difficult to resolve; thus, the refractory period is taken as the interval (c to d)

(B) Light-induced current (LIC; red) responses of the full stochastic model to a bright light pulse. Black dotted line highlights the summed response of the first bumps alone. Fast adaptation causes the decay of the initial sharp transient in a bright light pulse response. ~60% of fast adaptation is structural, caused by reduction in the number of activated microvilli, and the rest is by bump adaptation (see Figure 2G). Slow adaptation includes the slow decay to a plateau (as seen at the end of the light pulse in the red trace). These results were obtained by simulating 30,000 stochastic phototransduction cascades. In the model, slow adaptation was regulated by global negative feedback parameter, n_s, which increased exponentially with time (Table S2; see also Figure S3B).

(C) The width of the transient can be regulated by the bump latency, because the bump latency determines the jitter of the first bumps within microvilli population. Here, we summed up 30,000 “mock” bump series, using fixed values for the bump amplitude (1.8 pA), bump duration (51 ms), and refractory period (0 ms).

(D) *Drosophila* photoreceptors with variable degrees of dark-adaptation generate voltage responses to a bright blue-green pulse with variable transient widths. The width of the transient varies with past light exposure; the less dark-adapted the cell the narrower the transient. Use of intense blue light affects the rhodopsin:metarhodopsin ratio, at times inducing prolonged depolarizing after potentials (PDA; [3]).

(E) Voltage responses simulated by the combined model resemble *in vivo* recordings to the same stimulus; PDA is not modeled. Simulations show that the transition from prolonged dark-adaptation to light-adaptation [4, 5], which narrows the width of the early transient voltage responses, can be replicated by narrowing the bump latency distribution (see also Figure S3F) and understood as a memory of light exposure [6]; presumably mediated by intracellular calcium-accumulation affecting the microvilli globally.

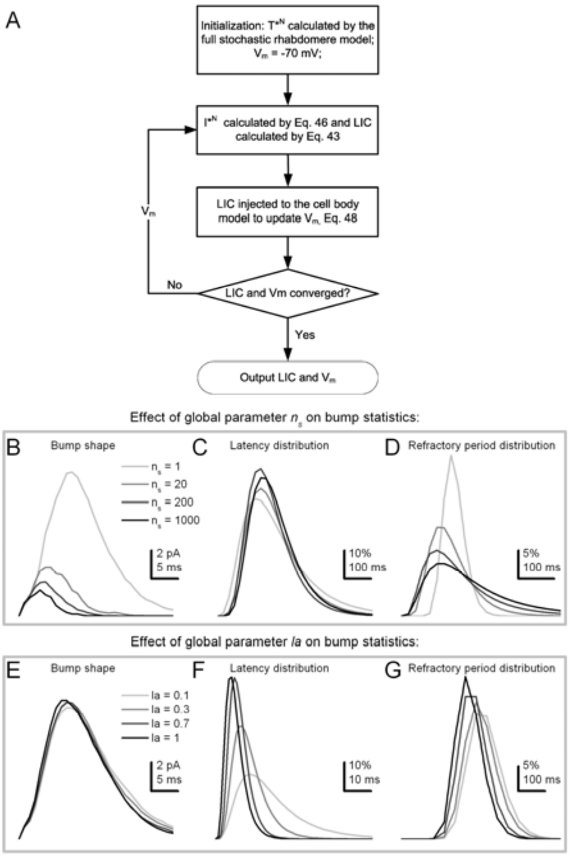

Figure S3. Global Regulation of Bump Parameters in the Simulations, Related to Figure 3

(A) Diagram showing how the global voltage feedback was calculated.

(B-D) Roles of the global feedback parameter, n_s, (Eq. 32) on bump shape, bump latency distribution and refractory period distribution. Note that in naturalistic stimulation experiments shown in Figure 4, n_s was always <20 while *la* was fixed. Therefore, in that range, brightening stimulation reduces the mean refractory period.

(B) n_s was used to adjust the bump shape in the simulations.

(C) Changing this parameter has much less impact on bump latency distribution.

(D) When bumps are made briefer, as used in the simulations at bright light levels, the tail of the refractory period distribution prolongs (peak shortens *cf*. dark-adapted). The overall range of these refractory period distributions agrees with the estimates obtained from *arr2*[5] mutant experimental data [7]. Latency and refractory period were used as defined in Figure 2A.

(E-G) Roles of the latency width-regulator parameter, *la*, (Eq. 33) on bump shape, bump latency distribution and refractory period distribution.

(E) *la* was used to adjust the bump latency distribution in the simulations.

(F) Changing *la* had relatively little impact on bump shape (0.1≤ *la* ≤1).

(G) Changing *la* had relatively little impact on bump refractory distribution (0.1≤ *la* ≤1).

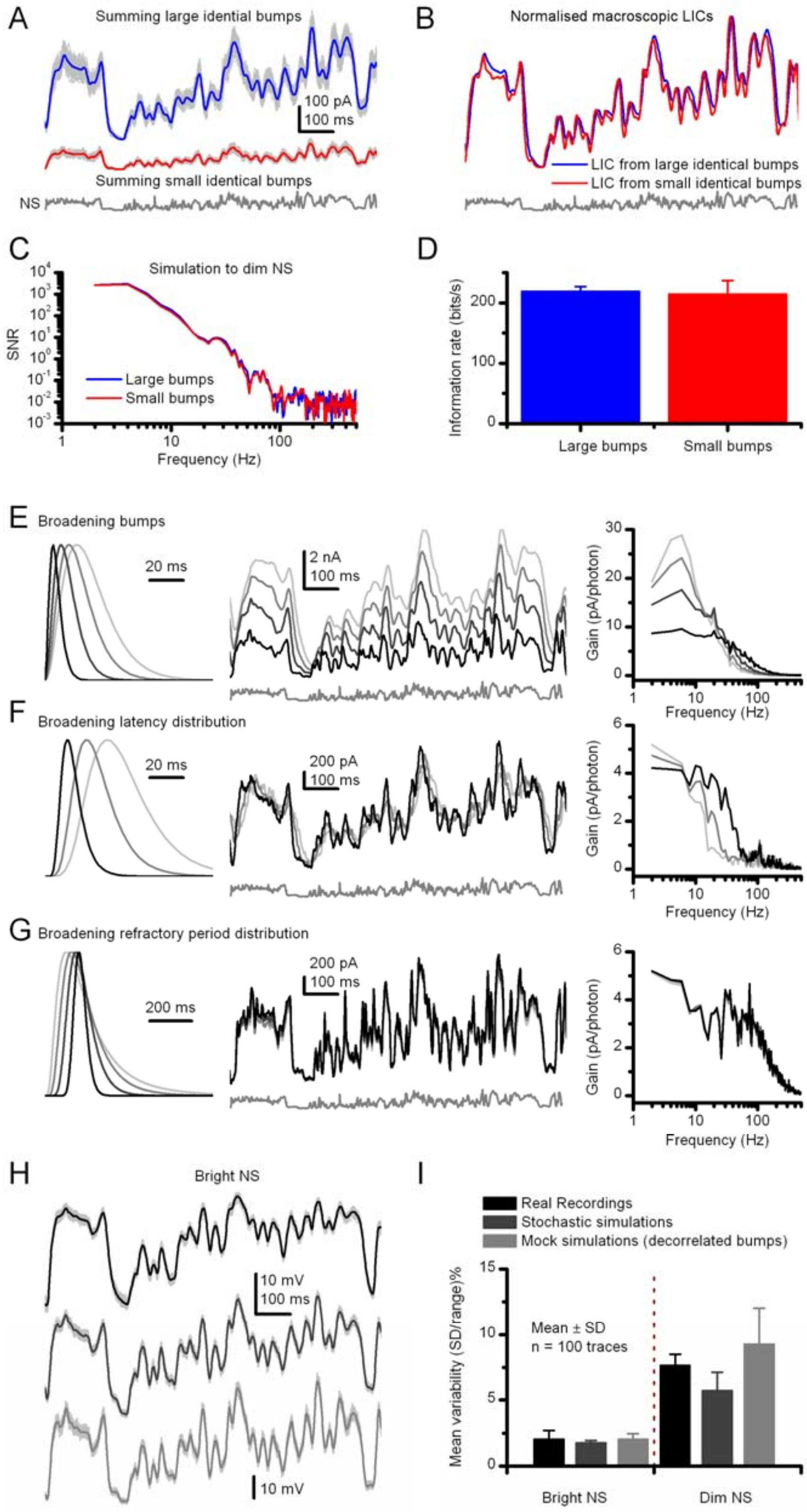

A
Summing large idential bumps
100 pA
100 ms
Summing small identical bumps
NS
B
Normalised macroscopic LICs
LIC from large identical bumps
LIC from small identical bumps
C
Simulation to dim NS
SNR
Large bumps
Small bumps
Frequency (Hz)
D
Information rate (bits/s)
Large bumps
Small bumps
E
Broadening bumps
20 ms
2 nA
100 ms
Gain (pA/photon)
Frequency (Hz)
F
Broadening latency distribution
20 ms
200 pA
100 ms
Gain (pA/photon)
Frequency (Hz)
G
Broadening refractory period distribution
200 ms
200 pA
100 ms
Gain (pA/photon)
Frequency (Hz)
H
Bright NS
10 mV
100 ms
10 mV
I
Real Recordings
Stochastic simulations
Mock simulations (decorrelated bumps)
Mean variability (SD/range)%
Mean ± SD
n = 100 traces
Bright NS
Dim NS

Figure S4. Related to Figure 5

Bump waveform does not affect the rate of information transfer; as long as the bumps are briefer than the stimulus changes they encode. But together with the stochastic latency and refractory period distributions, the bump waveform regulates the size (gain) and speed (frequency range) of macroscopic light-induced currents (LIC).

(A) LIC to dim naturalistic stimulation (NS) generated by mock-simulations, which integrated either identical large bumps or identical small bumps to dim naturalistic light intensity series (NS).

(B) The mean LICs appear similar when normalized, but as expected, the small bumps sum up current deflections with somewhat finer time and amplitude resolution.

(C) Signal-to-noise ratio (SNR) is equal for the both light current responses.

(D) According to the data processing theorem [8, 9], bump shape is a filter that affects signal and noise equally. Therefore, when having equal number of bumps (samples) of equal timing, the rate of information transfer of the two light current responses must be (and is) the same.

The two simulations in (A and B) were done by replacing each stochastically absorbed photon (at mean intensity of 3,000 photons/s) with either identical large or identical small bumps (Supplemental text, p. 20). The simulations ignore noise from sources other than bumps.

(E-G) Contribution of bump parameters to signal filtering.

(E) Broadening the bumps (left) increases the gain and low-pass filtering of the macroscopic responses to naturalistic light inputs (middle), reducing the bandwidth of linear frequency responses (right).

(F) Broadening the latency distribution (left) increases low-pass filtering of the macroscopic responses to naturalistic light inputs (middle), reducing the bandwidth of linear frequency responses (right).

(G) Broadening the refractory period distribution (left) has little effect on the filtering of the responses (middle) and their frequency range (right). In these simulations, both the bump shape and latency distribution were kept very brief, extending the gain to high stimulus frequencies. However, the broader the refractory period, the fewer absorbed photons generate bumps. This reduces the sample rate and, thus, the rate of information transfer.

The gain estimate of the linear transfer function between NS and the average light current was obtained by standard methods [5, 6], based on Eq. 1 (Supplemental text, p. 10).

(H) Real recordings (above) to bright NS compared to similar stochastic simulations (middle), and to mock stimulations, in which the bumps are decorrelated (below). Thicker traces show the means, thin light-gray traces show 100 individual responses. Note that real recordings contain also noise from damage, micro-saccadic eye movements [10], and molecular translocations [11-14]. In mock simulations, LIC was filtered by the HH-membrane model to approximate voltage responses.

(I) Comparison of the variability of the real recordings and stochastic and mock simulations to bright and dim NS. The variability in the stochastic simulations resembles that in the real recordings, both to the bright and dim NS, whereas the mock simulations (with decorrelated bumps) to dim NS are considerably noisier.

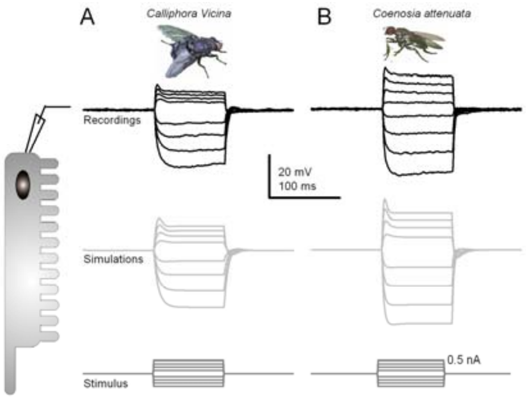

Figure S5. Membrane Properties of Blowfly and Killer Fly R1-R6 Photoreceptors, Related to Figure 6

(A, B) Voltage responses of real *Calliphora* and *Coenosia* photoreceptors, respectively (above), and those of their Hodgkin-Huxley photoreceptors membrane models (below) to injected current steps in darkness. In both cases, depolarizing current steps cause pronounced outward rectification due to activation of delayed-rectifier voltage-gated potassium channels.

Table S1. Summary of Fixed Phototransduction Parameters, Related to Figures 1-7

Parameter	Definition	Value
K_p	Dissociation coefficient for calcium positive feedback	0.3 mM (*tuned*)
K_n	Dissociation coefficient for calmodulin negative feedback	0.18 mM (*tuned*)
m_p	Hill constant for positive calcium feedback	2 [15]
m_n	Hill constant for negative calmodulin feedback	3 [15]
m	Co-operativity parameter of D* to TRP/TRPL channels	2 [15]
h_{M^*}	Strength of negative calmodulin feedback to M*	40 (*tuned*)
h_{PLC^*}	Strength of negative calmodulin feedback to PLC	11.1 [15]
h_{D^*}	Strength of negative calmodulin feedback to D*	37.8 [15]
$h_{T^*,p}$	Strength of positive calcium feedback to TRP/TRPL channels	11.5 (*tuned*)
$h_{T^*,n}$	Strength of negative calmodulin feedback to TRP/TRPL channels	10 (*tuned*)
κ_{G^*}	Rate of G* activation	7.05 s^{-1} (*tuned*)
κ_{PLC^*}	Rate of PLC* activation	15.6 s^{-1} (*tuned*)
κ_{D^*}	Rate of D* activation	1300 s^{-1} (*tuned*)
κ_{T^*}	Rate of TRP/TRPL channels' activation	150 s^{-1} (*tuned*)
K_{D^*}	Transition rate from D* to opening of TRP/TRPL channels	100 (*tuned*)
γ_{M^*}	Deactivation rate of M*	3.7 s^{-1} [15]
γ_{G^*}	Conversion rate from G_αGDP to G protein	3.5 s^{-1} (*tuned*)
γ_{PLC^*}	Deactivation rate of PLC*	144 s^{-1} (*tuned*)
γ_{D^*}	Deactivation rate of D*	4 s^{-1} (*tuned*)
γ_{T^*}	Deactivation rate of TRP/TRPL channels	25 s^{-1} (*tuned*)
γ_{GAP}	Deactivation rate of G-protein by GTPase activity	3 s^{-1} (*tuned*)
T_T	Total number of TRP/TRPL channels	25 [16]
G_T	Total number of G-protein	50 [17]
PLC_T	Total number of PLC	100 [16]
P_{Ca}	Percentage of Ca^{2+} in LIC	40% [18]
I_{T^*}	Average current through one opened TRP/TRPL channel	0.68 pA [4]
$[Na^+]_o$	Extracellular sodium concentration	120 mM [19]

$[Na^+]_i$	Intracellular sodium concentration	8 mM	[18]
$[Ca^{2+}]_o$	Extracellular calcium concentration	1.5 mM	[19]
$[Ca^{2+}]_{id}$	Intracellular calcium concentration in the dark	160 nM	[20]
$[C]_i$	Concentration of calmodulin inside a single microvillus	0.5 mM	[21]
n	Binding sites for calcium on calmodulin	4	[22]
F	Faraday constant	96,485 C/mol	
T	Absolute temperature	293 K	
R	Gas constant	8.314 $JK^{-1}mol^{-1}$	
K_U	Rate of Ca^{2+} uptake by calmodulin	30 $mM^{-1}s^{-1}$	[15]
K_R	Rate of Ca^{2+} release by calmodulin	5.5 s^{-1}	[15]
K_{Ca}	Rate of Ca^{2+} diffusion from microvillus to somata	1,000 s^{-1} (*tuned*)	
V	Microvillus volume	$3 \cdot 10^{-12}$ µl	
K_{NaCa}	Scaling factor for Na^+/Ca^{2+} exchanger model	$3*10^{-8}$ pA/mM^4	
C_m	Overall membrane capacitance (includes the cell body + rhabdomere)	62.8 pF	[1]

Where possible, the parameter values were taken from published estimates, with the corresponding references in parentheses. If the dynamics of underlying processes had not been characterized before (such as those for Ca^{2+} feedbacks), the parameter values were tuned to generate the appropriate bump statistics. It is possible that these tuned values may deviate from biophysical reality. However, these parameter values produced macroscopic responses that matched those of the experimental recordings. Thus, possible inaccuracies in the parameter values should not influence the general conclusions of this paper. In the simulations of this paper, all the parameters were fixed to the values of this table.

We further performed tests that probed the robustness and predictability of our simulated results:

(a) By comparing the results of mock simulations, in which stochastically generated bumps were replaced by large or small identical bumps, to those of actual stochastic simulations (Figures S4A-D); and further to the real recordings. (b) By comparing results of the stochastic simulations to those of the mock simulations, in which stochastically generated bumps were replaced randomly by bumps with similar amplitude and width distributions (Figure 5A). These tests verified that, as long as the tuned parameters generate bump production dynamics that mimic those seen in the given light conditions, the model outputs are realistic

Therefore, this computational approach, which combines intracellular electrophysiology with biophysical modeling, is very robust and does not require full knowledge of all molecular players and dynamics in the phototransduction. Indeed, whilst we have endeavored to adhere to the detailed molecular knowledge of fly phototransduction, from a computational viewpoint the exactness of the simulated molecular interactions is not important. As long as the photoreceptor model contains the right number of microvilli, each of which is a semi-autonomous sampling unit, and their stochastic bump dynamics (average waveforms, latency distribution and refractory period) approximate those in the real recordings, it will sample and process information like a real photoreceptor.

Table S2. Parameters Used for the Slow Adaptation; Exponential Decay, Related to Figure 2

Variable	Dim background	Bright background
n_{s0}	1	2
A_{n_s}	4	200
τ_{n_s}	3,000	1,000

n_{s0} is the initial condition of the global negative feedback parameter. A_{n_s} is the upper-bound for the dynamic increase in n_s. τ_{n_s} is the time constant of the negative feedback.

Supplemental Experimental Procedures

Patch-Clamp Recordings

For whole-cell photoreceptor recordings, dissociated ommatidia were prepared as previously described [23] from newly eclosed adult *w^{1118}* flies and transferred to the bottom of a recording chamber on an inverted Nikon Diaphot microscope. The bath contained (in mM): 120 NaCl, 5 KCl, 10 *N*-Tris-(hydroxymethyl)-methyl-2-amino-ethanesulphonic acid, 4 $MgCl_2$, 1.5 $CaCl_2$, 25 proline and 5 alanine, pH 7.15. The intracellular pipette solution was (in mM): 140 K gluconate, 10 *N*-Tris-(hydroxymethyl)-methyl-2-amino-ethanesulphonic acid 4 Magnesium-ATP, 2 $MgCl_2$, 1 NAD and 0.4 Sodium-GTP, pH 7.15. All chemicals were obtained from Sigma-Aldrich. Whole-cell voltage clamp recordings were made at room temperature (20 ± 1° C) at –70 mV (including correction for –10 mV junction potential) using electrodes of resistance ~10-15 MΩ. Series resistance values were generally below 30 MΩ and were routinely compensated to >80%. Data were collected and analyzed using an Axopatch 200 amplifier and pCLAMP10 software (Molecular Devices, Union City CA). Photoreceptors were stimulated via a green light-emitting-diode. Intensities were calibrated in terms of effectively absorbed photons in wild type photoreceptors by counting quantum bumps at low intensities.

Intracellular Recordings

We recorded voltage responses in R1-R6 photoreceptors of wild-type red-eye fruit flies (*Drosophila melanogaster*), blowflies (*Calliphora vicina*) and killer flies (*Coenosia attanuata*) to light and current stimuli, using sharp quartz microelectrodes (120–220 MΩ with 3 M KCl) fabricated with a Sutter Instruments P2000 puller. How to prepare flies for *in vivo* experiments and the general electrophysiological methods are explained in [5, 9, 24]. To minimize effects of damage and external noise, such as instrumental noise or extrinsic neural/muscle activity, on the analysis, only very stable recordings of low-noise and high sensitivity were selected for this study. Such photoreceptors had dark-adapted resting potentials <-60 mV and >50 mV responses to saturating light impulses.

Light Stimuli

Two light sources were used in the experiments. In Figure 4, which tested encoding of maximally bright stimuli (similar to full daylight), the light source was a high power light emitting diode (Seoul Z-Power LED P4 star, white, 100 Lumens) driven by an OptoLED (Cairn Research Ltd, UK) and fitted with a lens and a pinhole (subtended angle to 0.7°, as seen by the flies) [24]. In Figure 3, we used a cluster of high-power LEDs, in which output was scaled in $Log_{10}/2$ steps by a custom-built motorized neutral density filter wheel and focused on one end of a light guide [5, 9]. The light sources were attached onto a Cardan arm system, enabling exact positioning of the stimuli at the centre of a photoreceptors receptive field. Light stimuli (pulses, pseudorandom and naturalistic light intensity series) were generated and sampled together with voltage responses at 1-10 kHz using a National Instruments 12-bit A/D converter, controlled by a custom-written software system (Biosyst [5, 9, 24]) in Matlab environment. The stimuli and responses were filtered at 500 Hz (KEMO VBF/23 low pass elliptic filter), before these were sampled and stored to the hard-drive of the computer. The data was analyzed offline using Matlab.

Linear Impulse Responses

A predetermined luminance level (BG-5 to BG0) was switched on for 10 s, followed by repeated presentations (10-15 times) of a 10 s long pseudorandom contrast stimulus, $c(t)$, superimposed upon it. $c(t)$ had Gaussian amplitude distribution and a 'flat' power spectrum up to 200 Hz. It elicited similar response dynamics as reported earlier [5, 9, 24], without wasting much power on higher frequencies that these photoreceptors cannot follow.

Transfer function, $T(f)$, between the average response, or 'signal' $s(t)$, and the contrast stimuli, using their 1,024-points long spectral estimates, $S(f)$ and $C(f)$, respectively, is:

$$T(f) = \frac{\left\langle S(f) \cdot C^*(f) \right\rangle}{\left\langle C(f) \cdot C^*(f) \right\rangle} \tag{1}$$

Here <> indicate the average over the different stretches, and * the complex conjugate. The linear impulse response, or 1st order Wiener kernel, was obtained by taking the inverse Fourier transform of its frequency response:

$$k(t) = F^{-1}\left[T(f)\right] \tag{2}$$

Signal-to-Noise Ratio

1 s long naturalistic light intensity series (10,000 points), selected from van Hateren natural stimulus collection, was repeated over 100 times, and the evoked voltage responses of a photoreceptor were recorded. Because of their systematic adaptive trends, the first 5-20 traces were rejected from the analysis. For each recording series (Figures 4 and 6), the averaged response was the 'signal', while the 'noise' was the difference between individual traces and the 'signal'. Hence for an experiment using n trials (with n = 50-100) there is one 'signal' trace and n 'noise' traces.

For the analysis, the 'signal' and 'noise' traces were divided into 50% overlapping stretches and windowed with a Blackman-Harris 4-term window, each giving three 500-points long samples. As all the data from the 50-100 voltage traces was used, we obtained 150-300 spectral samples for the noise, and 3 spectral samples for the signal. These were averaged, respectively, to improve the estimates.

Signal-to-noise ratio SNR(f) of the voltage responses was calculated from the signal and noise power spectra, $\langle|S(f)|^2\rangle$ and $\langle|N(f)|^2\rangle$, respectively, as their ratio (Figure 4G), where | | denotes the norm and <> the average over the different stretches [5].

Bump and Latency Distribution Analysis

It has been demonstrated in different insect photoreceptors that at each light level (adapting background), or during continuous white-noise light contrast stimulation, the measured voltage noise of light-adapted photoreceptors, $|N(f)|^2$, contains mostly light-induced noise [5, 6, 25, 26] (i.e. bump noise: $|B(f)|^2$. Thus:

$$\left|B(f)\right|^2 \cong \left|N(f)\right|^2 \tag{3}$$

From the voltage noise power (Figure 3G), the effective bump duration, T_d, can be estimated [5, 6, 25-28]; assuming that the shape of the bump function, $b(t)$ (Figure 3I), is proportional to the Gamma-distribution □ (t;n, τ):

$$b(t) \propto \Gamma(t; n, \tau) = \frac{1}{n!\tau}\left(\frac{t}{\tau}\right)^n e^{-\frac{t}{\tau}} \tag{4}$$

This assumption has been shown to be true for single bumps [6]. The two parameters, *n* and *τ,* can be obtained by fitting a single Lorentzian to the experimental power spectrum of the bump voltage noise (Figure 3H):

$$|B(f)|^2 \propto \left|\tilde{\Gamma}(f;n,\tau)\right|^2 = \left[1+(2\pi\tau f)^2\right]^{-(n+1)} \quad (5)$$

Where $\tilde{\Gamma}$ indicates the Fourier transform. The effective bump duration, T_d (*i.e.*, the duration of a square pulse with the same power), is then:

$$T_d = \tau \frac{(n!)^2\, 2^{2n+1}}{(2n)!} \quad (6)$$

The brighter the light stimulus, the briefer the average bump duration is. In *Drosophila*, brightening reduces the bump duration from ~40 ms (dim background) to ~10 ms (bright background) [5, 25] (Figure 3I).

Because the estimated bump shapes, *b*(*t*), full-fill the minimum phase criteria [4, 5, 25, 29], the variance in timing of individual bumps, *i.e*. their latency distribution, *l*(*t*), can be estimated accurately from the voltage responses at different adapting backgrounds. This analysis greatly benefits from the linearizing effect, which white-noise (pseudorandom) contrast stimuli has on photoreceptor output. Therefore, based on the adapting bump model[30], we can assume that the underlying processes are linear and that a photoreceptor's impulse response, *k* (*t*), can be described as a convolution of the average bump waveform, *b* (*t*), and the distribution of bump latencies, *l*(*t*), for a given light intensity level.

$$k(t) = b\ (t) \otimes l(t) \quad (7)$$

Thus, latency distribution can simply obtained by deconvolving the bump shape out of the impulse response (Figure 3J).

The Rate of Information Transfer

The response of a photoreceptor in the voltage domain to naturalistic stimulation (NS) is nonlinear and non-Gaussian [9, 31]; for such conditions the classic Shannon formula for information capacity is not valid. Therefore, to calculate the rate of information transmitted by the photoreceptors, we used the triple extrapolation method, which has been shown to obtain robust estimates from continuous neural responses [9].

The voltage responses of the *Drosophila*, *Coenosia* or *Calliphora* photoreceptors were first digitized by dividing these into time intervals, T_w, that were subdivided into smaller intervals of t_w = 1 ms. This procedure selects 'words' of length T_w with T_w/t_w 'letters'. The mutual information between the voltage response *S* and the light contrast stimulus is then the difference between the total entropy, H_s:

$$H_S = -\sum_i P_S(s_i)\log_2 P_S(s_i) \quad (8)$$

where $P_S(s_i)$ is the probability of finding the *i*-th word in the response, and the noise entropy H_N:

$$H_N = -\left\langle \sum_{i=1} P_i(\tau)\log_2 P_i(\tau) \right\rangle_\tau \quad (9)$$

where $P_i(\tau)$ denotes the probability of finding the i-th word at a time t after the initiation of the trial. This probability $P_i(\tau)$ was calculated across trials of identical NS. The values of the digitized entropies depend on the length of the 'words' T, the number of voltage levels v (upsilon) and the *size* (as %) of the data file, $H^{T,v,size}$. The rate of information transfer was then obtained taking the following three successive limits:

$$R = R_S - R_N = \lim_{T_w \to \infty} \frac{1}{T_W} \lim_{v \to \infty} \lim_{size \to \infty} \left(H_S^{T_w,v,size} - H_N^{T_w,v,size} \right) \quad (10)$$

These limits were calculated by extrapolating the values of the experimentally obtained entropies. Because after removing the first trial (the first 5-20 traces when a trend caused by adaption could be seen), we typically used the next 100 traces (1 kHz sampling rate), thereby having a response matrix of 1,000 points x 100 trials for the analysis. The total entropy and noise entropy were then obtained from the response matrices using linear extrapolation with the following parameters:

size = 5/10, 6/10,…,10/10 of data; v = 6, 7,…,11 voltage levels; T^{-1}=3, 4,…,6 points.

Counting Microvilli in Rhabdomeres

We used published estimates for the number of microvilli in *Drosophila* and *Calliphora* R1-R6 outer photoreceptors [17, 32]. The number of microvilli in *Coenosia* R1-R6 photoreceptors was estimated using the published EM images, which provided the length of rhabdomeres in the retina and their transverse cuts [24] (giving the width of an average microvillus and the number of microvilli in a plane). These measurements gave the number of microvilli in rows and columns in the rhabdomere, and their product giving an estimate of the total number of microvilli. The number of microvilli was 30,000, 90,000 and 30,000 for *Drosophila*, *Calliphora* and *Coenosia* respectively.

Biophysical Model of *Drosophila* Photoreceptor

To simulate voltage responses of photoreceptors to light stimuli, we constructed a biophysical model that approximates the molecular reactions underlying phototransduction, $[Ca^{2+}]_i$ dynamics and the electrical response to ionic currents. The model was validated both in the current (*in vitro* voltage-clamp) and voltage (*in vivo* current-clamp) domains by comparing experimentally measured and simulated responses. The model was able to generate realistic responses to time series of various light intensities, including naturalistic stimuli, providing detailed dynamics of the key molecules. Therefore, these simulations could be examined to reveal and quantify biophysical mechanisms of light adaptation.

The model contains four modules (Figure S1A), reflecting the real structure and function of a *Drosophila* photoreceptor. The first module (i) is a random photon absorption model, which is based on Poisson statistics [33]; it governs the temporal history of photon absorptions in each microvillus. The second module (ii) models the chain of stochastic biochemical reactions in the phototransduction cascade inside a microvillus. It is assumed that 30,000 microvilli, which make up the rhabdomeres of a R1-R6 photoreceptor [34], capture and transduce the energy of photons independently. In the third module (iii), the sum of light-induced currents (LICs) from all the microvilli represents the macroscopic responses. The fourth module (iv) is a Hodgkin-Huxley model of the photoreceptor cell membrane [1, 2]; it transduces LICs into voltage responses.

(i) Random Photon Absorption Model

The input to this module is the number of photons absorbed by the rhabdomere at each time interval (1ms), while the output is the number of photons absorbed by each microvillus. Light intensities, used in the simulations, were calibrated using *in vivo* experiments in terms of average absorbed photons, by counting quantum bumps at low intensities. In the model, photons are absorbed randomly following Poisson statistics [33], leading to variable numbers of photons absorbed by each microvillus across the rhabdomere (Figure S1B). Importantly, because the LIC/photon ratio in each microvillus changes with the number of photons it absorbs, the random photon absorption model is crucial for producing a realistic light input for each microvillus.

We assume that each microvillus absorbs photons independently with all of them having equal absorption probabilities. This assumption simplifies calculations by eliminating the role of geometry in the microvillar array. In reality, the lengths of microvilli differ in the rhabdomeres; they shrink along the longitudinal axis and taper across the same section, being longest in the middle. It is reasonable to think that the shorter the microvillus, the less its photon absorption probability. Furthermore, their photon absorption probabilities at the bottom are likely to be less than those at the top of photoreceptor cell. Since the simulations matched the experimental results well, no further probabilistic scaling of the photon flux (through the tapering microvilli) was done here.

If the light input contains N_{ph} photons at the given time interval (1 ms), the average number of photons a single microvillus can absorb is $\lambda_M = N_{ph}/N_{micro}$, where N_{micro} = 30,000 denotes the number of microvilli in the rhabdomere. Following Poisson statistics, the fraction of microvilli that absorb k photons is $p(k) = \lambda_M^k e^{-\lambda_M} / k!$, so we can uniformly draw $p(k)N_{micro}$ microvilli to absorb k photons at this time point, where $k < k_n$ and k_n denotes the smallest number that satisfies $p(k_n) < 1/ N_{micro}$. This procedure iterates along the light time series; each microvillus then obtains an absorbed photon series to stimulate its phototransduction cascade.

(ii) Phototransduction Kinetic Model

The phototransduction cascade model is built upon known enzymatic reactions, modified from an earlier publication [15]. The major improvement of our bump model is that it has been extrapolated to accommodate sequential photon absorptions to generate bump series; the previous models generated only single quantum bumps (not series). This property is essential for studying continuous light adaptation processes. Furthermore, despite the many model parameters, adaptation mechanisms in our model were regulated by two parameters only: n_s in Eq. 32 for bump shape and *la* in Eq. 33 for bump latency distribution. This model also has a more realistic representation of $[Ca^{2+}]$ dynamics.

To account for the intrinsic randomness in the phototransduction reactions, where several molecular interactions occur at low numbers, the quantum bump model uses a stochastic simulation framework using the Gillespie algorithm [35]. The Gillespie algorithm is a rigorously derived Monte Carlo technique that is fully equivalent to the underlying Chemical Master Equation [35] (CME). It applies to a well-mixed chemical system (assumed here for simplicity), where all the reaction pathways are decomposed into M unidirectional elementary reaction steps R_μ. Each of these reaction steps is characterized by a momentarily-defined stochastic reaction constant, c_μ, where $c_\mu \delta t$ (μ = 1, 2, … , M) denotes the average probability that a particular combination of R_μ reactant molecules will react accordingly in the next infinitesimal time interval δt. If we define h_μ as the total number of R_μ reactant pairs, $a_\mu \delta t = c_\mu h_\mu \delta t$ is the average probability that reaction R_μ will occur during δt. By considering a discrete infinitesimal time interval, (t, $t+dt$), during which 0 or 1 reaction occurs, dt and R_μ can be determined independently. When R_μ is chosen, the state vector $\mathbf{X}$ is updated with a state transition vector, $\mathbf{V}_\mu$. This procedure iterates until a termination criterion is satisfied; for example, if the current simulation time, t, is larger than a preset value.

Following this framework, we will first describe the bump kinetics and then define the key parameters. The molecules, which are few in each microvillus, are counted, otherwise, we use concentrations (these two metrics are related by the microvillus volume V. In the following equations or reactions, X denotes the number of molecules, X^* denotes to the active state of X, and X_T denotes the total number of corresponding molecules/channels inside a single microvillus; $[X]$ denotes the concentration, $[X]_i$ denotes intracellular concentration, $[X]_o$ denotes the extracellular concentration. Rates of activation are generically denoted by κ and rates of deactivation denoted by γ. Positive and negative feedbacks are denoted by f_p and f_n, respectively and the local feedback strength parameter is denoted by h. Since spontaneous bumps are very rare, we assume that no reactions occur in the dark adapted state.

The signaling pathway is decomposed into a set of unidirectional reactions, each of which contains only unimolecular or bimolecular reactants:

$$M^* \xrightarrow{c1} \phi \quad \text{R1}$$

$$M^* + G \xrightarrow{c2} M^* + G^* \quad \text{R2}$$

$$G^* + PLC \xrightarrow{c3} PLC^* \quad \text{R3}$$

$$G^* + PLC^* \xrightarrow{c4} G_\alpha GDP + PLC^* \quad \text{R4}$$

$$G_\alpha GDP \xrightarrow{c5} G \quad \text{R5}$$

$$PLC^* \xrightarrow{c6} D^* + PLC^* \quad \text{R6}$$

$$PLC^* \xrightarrow{c7} PLC + G_\alpha GDP \quad \text{R7}$$

$$D^* \xrightarrow{c8} \phi \quad \text{R8}$$

$$2D^* + T \xrightarrow{c9} T^* \quad \text{R9}$$

$$T^* \xrightarrow{c10} T \quad \text{R10}$$

$$Ca^{2+} + CaM \xrightarrow{c11} C^* \quad \text{R11}$$

$$C^* \xrightarrow{c12} Ca^{2+} + CaM \quad \text{R12}$$

Since the light stimulus is already calibrated as the number of absorbed photons, photon activated rhodopsin (metarhodopsin, M^*) is incremented by 1 if one photon is absorbed in a microvillus. In the unidirectional reactions, R_1 denotes the inactivation of metarhodopsin (M^*) by arrestin binding; ϕ may indicate any reaction product, whose kinetics are not modeled. G-proteins can exist in multiple states. For simplicity, we only model the cycle through three states, $G_\alpha G_{\beta\gamma}GDP$ (G), $G_\alpha GTP$ (G^*) and $G_\alpha GTP$-PLC (PLC^*). R_2 represents the activation of G into G^* by M^*. G^* binds to PLC and becomes an active G-protein-PLC complex (PLC^*) in reaction R_3. Reactions R_4 and R_5 represent the recycling process of G-proteins. R_4 is the conversion from $G_\alpha GTP$ to $G_\alpha GDP$ by GTPase activity of G^*, supposedly catalyzed by PLC^*; $G_\alpha GDP$ then rebinds to $G_{\beta\gamma}$ before it can be reactivated (R_5). PLC^* hydrolyses PIP_2 into DAG and IP_3, generating the unknown excitation messengers for TRP/TRPL channels. Because of this ambiguity in the molecular mechanisms, we model in R_6 directly from PLC^* to the supposed excitation messenger D^* (*i.e. DAG*). D^* excites TRP/TRPL channels T to their open states (T^*) in reaction R_9 and is degraded in reaction R_8. Reaction R_{10} represents the process of closing the open TRP/TRPL channels. Reaction R_{11} represents the binding of Ca^{2+}, which enters the microvillus through the open TRP/TRPL channels, to Calmodulin C^*. Reaction R_{12} represents the release of Ca^{2+} from Calmodulin. The reaction R_{11} is a simplified 1st-order representation of the Ca^{2+} binding process, where in reality, 4 calcium ions bind to CaM to form C^*. Ca^{2+} bound Calmodulin constitutes the feedback intermediate

for regulating the reactions $R_1 - R_{10}$.

In these elementary reactions, *M** constitutes the activated metarhodopsin that has absorbed a photon and *T** constitutes the output for the phototransduction cascade. Under voltage-clamped condition, the light induced current is calculated from:

$$I_{in} = I_{T*} \times T* \tag{11}$$

where I_{T*} denotes the average single channel current conducted by an open TRP/TRPL channel.

Because we assume that apart from Ca^{2+} none of the molecular components can enter or leave the microvillus, the following mass balance equations hold:

$$T* + T = T_T \tag{12}$$

$$CaM + C* = C_T \tag{13}$$

$$PLC* + PLC = PLC_T \tag{14}$$

$$G*GDP + G + G* + PLC* = G_T \tag{15}$$

Using these mass balance equations the number of state variables can be reduced. Hence, the state vector is defined as:

$$\mathbf{X} = [M*; G; G*; PLC*; D*; C*; T*] \tag{16}$$

Ideally, Ca^{2+} should be included as one of the state variables. However, because Ca^{2+} changes up to 1,000-fold during a bump, its dynamics was approximated by a deterministic approach to save computational time. Hence, it was not included in the state vector (Eq. 16). How the Ca^{2+} dynamics was modeled is explained further down below.

According to R_1-R_{12}, the state transition matrix is defined as:

$$\mathbf{V} = \begin{bmatrix} -1 & 0 & 0 & 0 & 0 & 0 & 0 & 0 & 0 & 0 & 0 & 0 \\ 0 & -1 & 0 & 0 & 1 & 0 & 0 & 0 & 0 & 0 & 0 & 0 \\ 0 & 1 & -1 & -1 & 0 & 0 & 0 & 0 & 0 & 0 & 0 & 0 \\ 0 & 0 & 1 & 0 & 0 & 0 & -1 & 0 & 0 & 0 & 0 & 0 \\ 0 & 0 & 0 & 0 & 0 & 1 & 0 & -1 & -2 & 0 & 0 & 0 \\ 0 & 0 & 0 & 0 & 0 & 0 & 0 & 0 & 0 & 0 & 1 & -1 \\ 0 & 0 & 0 & 0 & 0 & 0 & 0 & 0 & 1 & -1 & 0 & 0 \end{bmatrix} \tag{17}$$

We can further derive the number of reactant pairs for each reaction:

$$\mathbf{h} = [M^*; (M^*)(G); (G^*)(PLC_T - PLC^*); (G^*)(PLC^*); \\ G_T - G^* - G - PLC^*; PLC^*; PLC^*; D^*; \\ \frac{(D^*)(D^*-1)(T_T - T^*)}{2}; T^*; (Ca^{2+})(CaM); C^*] \tag{18}$$

The stochastic reaction constant vector $\mathbf{c}_{1\times 12}$, is calculated from:

$$c1 = \gamma_{M^*}(1 + h_{M^*} f_n) \tag{19}$$

$$c2 = \kappa_{G^*} \tag{20}$$

$$c3 = \kappa_{PLC^*} \tag{21}$$

$$c4 = \gamma_{GAP} \tag{22}$$

$$c5 = \gamma_G \tag{23}$$

$$c6 = \kappa_{D^*} \tag{24}$$

$$c7 = \gamma_{PLC^*}(1 + h_{PLC^*} f_n) \tag{25}$$

$$c8 = \gamma_{D^*}(1 + h_{D^*} f_n) \tag{26}$$

$$c9 = \frac{\kappa_{T^*}(1 + h_{T^*,p} f_p)}{(K_{D^*})^2} \tag{27}$$

$$c10 = \gamma_{T^*}(1 + h_{T^*,n} f_n) \tag{28}$$

$$c11 = \frac{K_U}{V^2} \tag{29}$$

$$c12 = K_R \tag{30}$$

where K_{D^*} is a normalization constant from D^* to TRP/TRPL, K_U and K_R are rate constants for Ca^{2+} binding and release to/from Calmodulin. $c11$ is scaled from K_U by a factor V^2, because the deterministic model for Ca^{2+} dynamics is described in terms of concentrations, instead of number of molecules (Eq. 35).

Positive feedback f_p is formulated as a Hill function of $[Ca^{2+}]_i$ inside a microvillus:

$$f_p([Ca^{2+}]_i) = \frac{([Ca^{2+}]_i / K_P)^{m_p}}{1 + ([Ca^{2+}]_i / K_P)^{m_p}} \tag{31}$$

Where K_p denotes the dissociation constant, *i.e.*, $[Ca^{2+}]_i$ that provides half occupancy of Ca^{2+} binding sites for the channels. m_p is the Hill coefficient, describing the cooperativity of Ca^{2+} in exciting the channels.

Negative feedback f_n is formulated as proportional to a Hill function of $[C^*]_i$:

$$f_n([C^*]_i) = n_s \times \frac{([C^*]_i / K_n)^{m_n}}{1 + ([C^*]_i / K_n)^{m_n}}$$

(32)

where n_s denotes a global negative feedback strength parameter. This is one of the most important parameters in our model; it is the only parameter that is used to regulate the bump shape in our simulations (Figure S3B), whereas all the other kinetic parameters are fixed. K_n denotes the dissociation constant and m_n denotes the Hill coefficient for $[C^*]_i$. In reality, the affinity of $[C^*]_i$ might vary for different feedback targets, leading to different parameter values for K_n and m_n . However, for simplicity, we assume that the whole pool of available C^* binding sites have the same affinity properties and local feedback strengths are parameterized by h_*. This simplification provides a practical initial approximation, in absence of more complete mechanistic knowledge concerning the different underlying processes. The parameters (κ_*, γ_* and h_* , etc) are listed in Table 1.

With the definitions of ***V***, ***c*** and ***h***, the time increment *dt*, during which the next reaction R_μ reacts, is determined as:

$$dt = \frac{1}{la + a_s} \ln(\frac{1}{r_1})$$

(33)

where r_1 denotes a uniformly distributed random number. a_s denotes the dot product between ***c*** and ***h*** ($a_s = \sum_{\mu=1}^{\mu=M} c_\mu h_\mu$). Apart from the parameter n_s in Eq. 32, *la* (or latency width-regulator) is the only other critically important parameter in our model, affecting the latency distribution (Figure S3F). R_μ can be chosen in a way so that Eq. 34 satisfies:

$$\sum_{v=1}^{\mu-1} a_v < r_2 a_s \le \sum_{v=1}^{\mu} a_v$$

(34)

where r_2 is a uniformly distributed random number, and a_v is a product between c_v and h_v.

Calcium Dynamics

Ca^{2+} is an extremely important feedback signal in the phototransduction cascade. However, because of the vast number of calcium ions involved, the computation is too time-consuming to simulate via Gillespie algorithm. To streamline computations, we adopted the use of hybrid techniques [36-40], where Ca^{2+} dynamics was simulated by deterministic methods:

$$\frac{d[Ca^{2+}]_i}{dt} = \frac{I_{Ca,net}}{2VF} - n\frac{d[C^*]_i}{dt} - K_{Ca}[Ca^{2+}]_i$$

(35)

Eq. 35 models Ca^{2+} dynamics as a balance between net Ca^{2+} influx (first term), Ca^{2+} uptake by calcium buffer (second term) and Ca^{2+} diffusion to the cell body. *V* denotes the microvillus volume, *F* denotes the Faraday constant. *n* denotes the number of Calmodulin Ca^{2+} binding sites. $1/K_{Ca}$ denotes Ca^{2+} diffusion time constant. $I_{Ca,net}$ denotes the net Ca^{2+} influx (pA), which is a balance between Ca^{2+} influx, I_{Ca} and Ca^{2+} extrusion through Na^{+}/Ca^{2+} exchanger, $2I_{NaCa}$:

$$I_{Ca,net} = I_{Ca} - 2I_{NaCa} , \tag{36}$$

where I_{Ca} is estimated to constitute 40% of I_{in}. I_{NaCa} is calculated from Eq. 37, which is a simplified format of the Na^{+}/Ca^{2+} exchanger model [41], given that the extracellular ionic concentrations are fixed and the cell is voltage clamped:

$$I_{NaCa} = K_{NaCa}\left(\left[Na^{+}\right]_i^3 \left[Ca^{2+}\right]_o - \left[Na^{+}\right]_o^3 \left[Ca^{2+}\right]_i e^{-\frac{V_m F}{RT}} \right) \tag{37}$$

where K_{NaCa} denotes scaling factors; V_m denotes the transmembrane potential in Volts, approximated by resting potential (-70 mV); *R* denotes the gas constant and *T* denotes the absolute temperature.

Consistent with R_{11}—R_{12}, $\frac{d[C^*]_i}{dt}$ is defined as:

$$\frac{d[C^*]_i}{dt} = K_u([Ca^{2+}]_i)([CaM]_i) - K_R[C^*]_i \tag{38}$$

(Recall: K_U and K_R are the rate constants for Ca^{2+} uptake and release, respectively).

To update Ca^{2+} quantities in the stochastic simulation framework, Ca^{2+} dynamics are assumed to be so fast that the quantities can be approximated by the steady-state values. Hence, at each time point, based on Eq. 35, Ca^{2+} is calculated from:

$$\frac{d[Ca^{2+}]_i}{dt} = \frac{I_{Ca,net}}{2VF} - n\frac{d[C^*]_i}{dt} - K_{Ca}[Ca^{2+}]_i = 0 \tag{39}$$

Substituting Eq. 37 and Eq. 38 into Eq. 39, we obtain the steady-state Ca_{ss}^{2+} as:

$$Ca_{ss}^{2+} = V \times \frac{\frac{I_{Ca}}{2VF} + nK_R[C^*]_i - f_1([Na^{+}]_i,[Ca^{2+}]_o)}{nK_u[CaM]_i + K_{Ca} - f_2(V_m,[Na^{+}]_o)} \tag{40}$$

where $f_1([Na^{+}]_i,[Ca^{2+}]_o)$ is defined as:

$$f_1([Na^+]_i,[Ca^{2+}]_o) = K_{NaCa}\frac{[Na]_i^3[Ca^{2+}]_o}{VF} \quad (41)$$

and $f_2(V_m,[Na^+]_o)$ is defined as:

$$f_2(V_m,[Na^+]_o) = K_{NaCa}\frac{\exp(-\frac{V_mF}{RT})[Na^+]_o^3}{VF} \quad (42)$$

Simulating Phototransduction Model

A key new feature of our stochastic bump model is that it has been extended to accommodate complex time series of photon inputs. The idea is to stop the Gillespie algorithm and update the molecule numbers when a new photon input arrives.

The simulation procedure is as depicted in Figure S1C:

1. Initialize $\boldsymbol{X_0}$, $\boldsymbol{V}$, $\boldsymbol{h}$, $\boldsymbol{c}$, $\boldsymbol{a}$, t_{end}, T_{ph}, i=1, where $\boldsymbol{X_0}$ is the initial state vector, $\boldsymbol{V}$ is the state transfer matrix, $\boldsymbol{h}$ is the reactant pair vector, $\boldsymbol{c}$ is the stochastic reaction constant vector, $\boldsymbol{a}$ is the reaction propensity vector, t_{end} is the termination simulation time, T_{ph} is a vector recording the time point when there is photon stimuli, and i is iteration index for T_{ph}. N_{Rh} is the number of photon activated rhodopsins; here, the 4th element ($\boldsymbol{X_4}$) of the state vector, $\boldsymbol{V}$.
2. Generate two uniformly distributed random numbers: r_1 and r_2.
3. Decide the time increment for next reaction: $dt = \frac{1}{la + a_s}\ln(1/r_1)$, where $a_s = \sum_{\mu=1}^{\mu=M} c_\mu h_\mu$.
4. If $t+dt>T_{ph}(i)$, then let $t = T_{ph}(i)$, $i = i+1$, $N_{Rh} = N_{Rh} + N_{ph}(t)$, otherwise let $t = t+dt$.
5. Select R_μ that reacts during dt. R_μ is chosen so that $\sum_{v=1}^{\mu-1} a_v < r_2 a_s \le \sum_{v=1}^{\mu} a_v$ satisfied.
6. Update state vector $\boldsymbol{X} = \boldsymbol{X}+\boldsymbol{V_\mu}$, and update $\boldsymbol{h}$, $\boldsymbol{c}$ and $\boldsymbol{a}$ accordingly.
7. Update Ca^{2+} dynamics according to Eq. 39-42
8. If $t > t_{end}$, then stop simulation and output $\boldsymbol{X(t)}$, otherwise go back to 2.

Simulations of this extended bump model clarify why a single microvillus can only produce one bump at a time, regardless of its photon input (detailed explanations in the main text). Furthermore, the simulations also demonstrate how bumps adapt in a single microvillus, depending on its memory state of the photon input history. Importantly, the simulated bump series could be quantified by four bump parameters, namely: bump waveform (*i.e.* its amplitude and duration), latency distribution and refractory period distribution (Figure S2A). These bump parameters have great impact on the shape of the macroscopic LIC, whereas the model simulation enables an insightful link between the microscopic bump parameters and the macroscopic responses.

(iii) Integration of Light-Induced Current (LIC)

Role of Bump Parameters on Macroscopic Response

Individual microvilli only produce one bump at a time and the macroscopic current of the rhabdomere is integrated from the response of up to 30,000 microvilli. Although it is still unclear how different microvilli cooperate in producing LIC, we assume that their

phototransduction cascades are independent and the macroscopic LIC represents the summation of bumps (Eq. 43). This assumption is valid in dim to medium light conditions, where experimentally obtained bump statistics indicate one-to-one photons-to-bumps relationship [4].

$$LIC = \sum_{N=1}^{N=30,000} I_{in}^{N} \tag{43}$$

Given this assumption, how do the bump parameters affect the macroscopic response? To obtain better understanding of the bump parameters' interdependency, we also built a simplified bump integration model, which provided us quick information how the parameters shaped the macroscopic responses. For example, with LIC representing the integration of 30,000 'mock' bump series, we could examine the shape changes in LIC when only a single bump parameter was varied at a time.

Here, 'mock' bump series were not obtained through the phototransduction cascade model but instead these were generated separately, following several simple assumptions: each bump had a fixed shape, approximated by the gamma-function (Eq.34) [5]; the bumps were generated after a predetermined latency (stochastic or of prefixed value); no bumps were allowed to emerge in the middle of an ongoing bump response or during its refractory period; the refractory periods had either fixed values or these were generated for predefined distributions; the macroscopic response summed all bump series. In the simulations, three bump parameters were fixed, while the fourth one was changed to investigate its role.

$$I(t) = A\frac{e^{p}}{p^{p}}e^{-\frac{t}{\tau}} \tag{44}$$

where A, p, τ denote free parameters. We varied the bump amplitude with A and the bump duration with τ.

We considered responses to 2 s uniform square bright light pulse (3×10^5 photon/s), and to 1-2 s naturalistic stimuli. In both cases, the photon input to each microvillus was generated by the random-photon-absorption model.

How the mock simulations were done in key Figures:

The simulations using light steps indicated that:

1. The amplitude of the macroscopic LIC is determined by the bump shape; that is by the bump amplitude and duration. Both peak and plateau amplitudes increase proportionally with the bump amplitude, but sub-linearly with the bump duration.
2. The bump shape contributes the most in determining the LIC amplitude.
3. The transient width of LIC is determined by the bump latency (distribution) (Figure S2B).
4. The plateau level can be reduced by prolonging the refractory period, but long refractory periods induce oscillation.
5. Stochasticity in latency and refractory period benefits coding by expanding the input range and by reducing oscillations (Figure 5B).

The simulations using naturalistic stimuli indicated that:

6. Broadening the bumps (Figures S4E) or their latency distribution (Figures S4F) increased the low-pass filtering of the macroscopic responses,

7. Broadening the refractory period distribution had relatively little impact on a photoreceptor's encoding (Figures S4G). This is because naturalistic stimuli (of 1/*f* statistics) contain many dark or dim periods, which enable a large fraction of used microvilli to recover for the next bright light pattern. Thus, in comparison to light pulses, it is very difficult to saturate a system with naturalistic stimuli, which has over 30,000 independently operating microvilli (Figure 5D).

8. By shortening the refractory period, the availability of microvilli increases.

9. Stochasticity of the refractory period has relatively little influence on the overall response waveforms to naturalistic stimuli (Figure S4G), but it whitens the utilization of both the light current range and microvilli (Figure 5C) providing efficient usage of them.

Mock-Simulations (Large Bumps vs. Small Bumps) in Figures S4A-D. The following procedures and assumptions were used when generating bump series by 'mock' simulations: (a) each bump had a fixed shape; the large bump was the average of all bumps in the real stochastic simulation at dim light condition and the small bump was the average of all the bumps at bright light condition. (b) The bumps were generated after a predetermined stochastic latency, which followed the same latency distribution. (c) No bumps were allowed to emerge in the middle of an ongoing bump response, or during its refractory period. (d) The refractory periods were generated for the same predefined distribution, which was the refractory period distribution obtained from real stochastic simulations at dim light condition. (e) The macroscopic response was generated by summing all bump series.

Decorrelating Mock-Simulations for Small Bumps in Figure 5A and Large Bumps in Figure 5A. The amplitude and duration distributions of bumps to dim and bright naturalistic stimulation were first obtained from the respective stochastic simulations. In 'mock' simulations, the bumps' amplitudes and durations were randomly generated according to these distributions (for dim and bright simulations separately). This decorrelation added noise to the simulations (Figure S4I). Latencies and refractory periods were generated according to their own distributions, as obtained from the real stochastic simulation. The macroscopic response was generated by summing all bump series.

Fixed Refractory Period Mock-Simulations in Figures 5B-C. (a) All bump parameters and their corresponding distributions were predefined and were obtained from real stochastic simulations at bright light condition. (b) Each bump had a fixed shape, the average of all bumps. (c) The bumps were generated after the predetermined stochastic latency. (d) No bumps were allowed to emerge in the middle of an ongoing bump response or during its refractory period. (e) The stochastic refractory period distribution was obtained from the real stochastic simulation at bright light condition, while the fixed refractory period was set be 121 ms, which is the refractory period at the peak of the distribution. (e) The macroscopic response was generated by summing all bump series.

Model Parameter Adjustments

The bump waveform was regulated by a single parameter, n_s; this value was fixed for each simulation (and was not changed during the simulation). Similarly, the bump latency distribution was regulated by another single parameter, *la*, which was fixed for each simulation (and was not changed during the simulation). In extensive test simulations, we found that n_s affects mostly the bump shape (Figures S3B-D), whereas *la* affects mostly the bump latency (Figure S3E-G). Both of these parameters have little effect on the bump refractory period when the bump statistics were within the physiological range for *Drosophila* (Figures S3D and S3G).

(iv) HH Cell-Body Model

Drosophila R1-R6 Photoreceptor Membrane Model

To relate LICs to voltage responses, we adopted a Hodgkin-Huxley (HH) type photoreceptor cell membrane model, which incorporates voltage-gated fast inactivating Shaker and slow delayed rectifier, Shab, K^+ conductances [42, 43], and K^+ and Cl^- leaks. It also includes a slowly activating, non-inactivating voltage-gated K^+ conductances and the "window current" representing partial failure of Shaker channel inactivation (-11%). Further modulation of Shab channels, which are probably located on the microvillar plasma-membrane, via phosphoinositide depletion was not modelled [44]. The model also ignored the fast delayed rectifier (Shal current). As in earlier studies [1, 2], the conductance for Cl^- leak was used to regulate the resting potential. However, the light-dependent conductance is not included here, because the phototransduction cascade model provides the functional equivalence to this conductance. The equivalent electrical circuit of the cell body is shown in Figure S1D.

Blowfly and Killerfly R1-R6 Photoreceptor Membrane Models

Unlike *Drosophila* photoreceptors, blowfly (*Calliphora vicina*) outer photoreceptors do not have fast inactivating Shaker conductances [45]. So we exclude gksh from the model (Figure S1D) and regulate the other model parameters to approximate the voltage output of real blowfly photoreceptor membrane (Figure S5A). Although we do not know the exact potassium-channel composition in the *Killerfly* (*Coenosia attenuata*) photoreceptor membrane (Figure S5B), based on its voltage rectification to injected current-pulses [24], we assumed that its ion-channel composition resembles that of the *blowfly*. This view is supported by the general observation that photoreceptors of fast-flying flies seems to lack a fast inactivating Shaker-like membrane conductance [46]. Importantly, we further point out that in all insect photoreceptor membranes, which we have studied systematically both in dark- and light adapted conditions [5, 6, 24, 25, 47], the band-width of the cell-body membrane has always been much broader than that of the corresponding phototransduction cascades. Therefore, the photoreceptor membrane acts effectively as an adjustable scalar during naturalistic light stimulation, not influencing the transmitted information [9, 48]. By injecting the simulated macroscopic LIC to the cell body models of blowfly and killerfly photoreceptor membranes, we obtained the corresponding voltage responses, as shown in Figure 6 in the main paper.

(v) Global Feedback Mechanisms

To replicate the effect of slow global feedbacks, such as intracellular accumulation or diffusion of Ca^{2+}, we implemented a global feedback strength parameter in the phototransduction cascade model (n_s in Eq. 32). In the simplest case, by changing n_s exponentially (Eq. 45), the model reproduced the slow light adaptation trends (exponential decay to plateau) in the LIC responses to light pulses (Figure 2F).

$$n_s = n_{s0} + A_{n_s} - A_{n_s} \exp(-t / \tau_{n_s}) \tag{45}$$

where n_{s0} denotes the initial condition for n_s and $n_{s0} + A_{n_s}$ is the value for n_s at t_∞, τ_{n_s} denotes the time constant of n_s dynamics. These parameters for n_s at different light level are listed in Table.1. Since n_s impacts mostly bump shape, we conclude that the slow light adaptation is mainly caused by bump adaptation.

In simulations for Figures 3, 4 and 6 in the main paper, n_s parameter included no trend adjustment. This is because the output of the real photoreceptors, collected for the information analysis, were fully adapted to the repeated naturalistic input statistics (typically >20 s after the stimulus onset). Thus, these photoreceptors were operating in a steady-state

mode where slow light-adaptation played little role in their output dynamics. Notice that because of the abundance of microvilli, after the photoreceptor has adapted to a relative steady-state, its refractory period (distribution) remains brief enough to have very little (or no) effect on dynamic encoding of changing naturalistic light patterns (Figures S4G). Therefore, by fixing the bump shape and latency distribution in the stochastic models of different photoreceptors (*Drosophila*, *Calliphora* or *Coenosia*) to match those of the experimentally measured values (see **Bump and Latency Distribution Analysis**, above) at each mean light intensity level in real cells was sufficient to generate realistic voltage responses to naturalistic light intensity changes (Figures 3, 4 and 6). In other words, the stochastic model operated normally by dynamically adjusting the ratio of the used/available microvilli.

Owing to our model structure, the voltage regulation of TRP/TRPL channels' driving force was also implemented as a global feedback mechanism. This procedure allowed us to characterise the interplay between the light-sensitive and light-insensitive membrane, when the photoreceptor was encoding vast changes in light intensity.

Specifically, we modified I_{in} formulation from Eq. 11 to Eq. 46:

$$I_{in}^{N} = (T*^{N})(g_{TRP})(TRP_{rev} - V_m) \tag{46}$$

where $T*^N$ denotes the number of opened TRP/TRPL channels in the Nth microvillus, g_{TRP} is the single TRP channel conductance, TRP_{rev} is TRP channel reversal potential and V_m is the membrane voltage. g_{TRP} can be formulated according to Eq. 47.

$$g_{TRP} = 8 \times \begin{cases} 1 & if \quad TRP_{rev} > V_m \\ 0 & otherwise \end{cases} \tag{47}$$

In this way, TRP/TRPL channels are either fully closed or fully opened, with a single channel conductance of 8 pS at open state. TRP_{rev} is set to 0 mV[34].

From Eq. 46, as membrane voltage (V_m) increases, the bumps (I_{in}^{N}) shrinks accordingly, reducing the overall macroscopic LIC response (Eq. 43). Since the phototransduction cascades and the membrane are modeled separately in two compartments, the cell membrane voltage (V_m) is obtained by injecting LIC into HH-type cell body model:

$$V_m = HH(LIC) \tag{48}$$

where HH denotes the cell membrane model as a function.

The iteration process was conducted between these two model compartments (Eq.43 and Eq. 43-47) to obtain realistic macroscopic LICs and voltage responses. Thus, the calculation of I_{in}^{N} in Eq.46 requires membrane potential feedback, V_m, which is initialized at the resting potential (-70 mV). As a result, I_{in}^{N} in every microvillus is larger than the true value, and hence LIC_0 obtained in Eq. 43 is larger than the true value of *LIC*. Accordingly, LIC_0 charges a too large voltage response V_{m1}. To obtain a more realistic *LIC*, V_{m1} is fed back to Eq. 46 (Figure 3C); but as a result, LIC_1 can be smaller than the true value (*i.e.* the obtained values oscillate). Hence, an iterative simulation procedure between rhabdomere

and the cell body is conducted to acquire the converged *LIC* and V_m. In this process, it is assumed that the voltage feedback only affects the amount of current influx through the opened TRP/TRPL channels, but not the dynamics of the phototransduction; T^{*N} is not changing in each microvillus.

The simulated macroscopic LICs with and without the global voltage feedback are compared in Figure 3C. Thus, voltage acts as a global gain regulator, making all microvilli dependent on each other's activity. The brighter the stimuli, the more apparent this effect is.

Simulating Encoding in Steady-State Adapted Conditions

In **Figures 4** and **6**, we study encoding of naturalistic light contrasts at "steady-state" light-adapted conditions, *i.e.* when the slow bump adaptation to the given average light level has more-or-less settled out. To approximate this situation by the simulations, our naturalistic stimulus consisted of two identical one-second-long light intensity series patterns (*cf*. Figure 5D; NS, each having 10,000 points) taken from the van Hateren natural image collection [31]; the second pattern continued directly after the first one without any delay. We then simulated 100 responses of the full stochastic model to this stimulus. To eliminate the slow adaptive trend in the analyzed output, we typically only used the simulated responses to the second NS pattern (1001:2000 ms). Nonetheless, in Figure 5D (inset), we show the combined stimulus and the resulting microvilli usage for different brightness of NS over the whole stimulus duration.

The simulations clarify that the rate of information transfer of macroscopic voltage responses to naturalistic stimuli approaches a constant rate at bright light levels. There are three reasons for this. First, proportionally more photons hit rhodopsin-molecules in the microvilli during their refractory period, *i.e.* when they are unavailable for generating new bumps; thus, quantum efficiency drops. Concomitantly, the dynamic equilibrium between used and available microvilli (unused and recovering from their previous bumps; see also ***Negative feedback and refractory period***, below); approaches a constant (maximum) bump production rate (sample rate) (Figures 5B and 7D). Second, the global calcium and voltage feedbacks cannot make bumps any smaller and briefer with increasing brightness (*cf*. Figures S4E and 7A). Third, the bump latency distribution remains practically invariable in different light-adaptation states (Figure 3J). Therefore, when the sample rate and the filtering properties of the integrated macroscopic voltage responses settle (at intensities $>10^5$ photons/s in *Drosophila* R1-R6 photoreceptors), allocation of visual information in the photoreceptor's amplitude and frequency range becomes nearly invariable. Accordingly, the real recorded voltage responses of *Drosophila* R1-R6 photoreceptors in Figures 4D and 6A are very similar - and so are their rates of information transfer (see also [24, 49, 50])-, although we used much brighter ultra-bright LED stimulation in the latter case. As long as the microvilli usage is over 50% (Figure 5D), the rate of information transfer of the voltage responses become similar; and based on the ultra-bright naturalistic stimulation in these experiments, this is so even at very bright intensities.

Notice also that adaptation to brightening light intensity reduces the average bump duration in fly photoreceptors (Figure 3I), and that the estimated light-adapted bump latency distributions of *Calliphora* and *Coenosia* photoreceptors are much briefer than that of *Drosophila* (Figure 6A-C). In their respective photoreceptor models, when using physiological plausible range of the global bump parameters, n_s and l_a (*cf*. Figure S3B-G), these changes have an effect of shortening the bump refractory period distribution (Figure 6A-C; yellow). Therefore, the bump production rates (sample rates) of *Calliphora* and *Coenosia* microvilli during very bright simulation are higher than those of *Drosophila* for the same light stimuli. Naturally, the very brief bumps and latency distributions of *Calliphora* and *Coenosia* photoreceptors improve the capture of high-frequency information in 1/f stimuli; *i.e.* this information cannot be encoded by bumps and latencies of *Drosophila* photoreceptors that are slower than these high-frequency inputs themselves.

(iv) Highlighted Modeling Considerations

Negative Feedback and Refractory Period

Note that the time window for the general negative feedback parameter (calcium-calmodulin complex) $[C^*]_i$ decay phase is much longer than the refractory period, which is realistic and can be explained by two reasons. First, the negative feedback (f_n in Eq. 22) would have shorter decay phases as a Hill function of $[C^*]_i$. Second, a bump can be generated without C^* decaying to zero, as illustrated in the 3rd bump in Figure 1C. This is because the refractory period represents a balance between the positive and negative feedbacks, where the positive feedbacks can outgrow the negative feedbacks in the middle of $[C^*]_i$ decay process. In the models, Hill function was used for simplicity; assuming that the feedback functioned through a binding process, as there are Ca^{2+} and calmodulin binding sites on the ion channels.

Defining the Number of Activated Microvilli and Their Usage

To illustrate the role of stochastic sampling by microvilli, the total number of responding/activated microvilli (N_{villi}) was counted in 1 ms time-bins; *i.e.*, for $m = 1, 2, ..., 30,000,$ if $TRP^{*m} > 0$, $N_{villi}(t) = N_{villi}(t)+1$ (*e.g.* Figure 2). The temporal activation profile of N_{villi} matches well the photoreceptor voltage responses to naturalistic stimuli. To illustrate the role of stochastic refractory periods in the adaptive sampling, we further counted the number of used microvilli, *i.e.,* microvilli that are either responding or refractory. This counting was done by the simple bump integration model, where bump series were generated according to several simple assumptions (details in section **iii**, page 20).

Energy Costs Related to Adaptive Information Sampling

By modeling photoreceptors of different fly species and comparing their outputs to those of their real counterparts *in vivo*, we showed why and how their structure and biophysical makeup are intrinsically linked to the neural signaling they carry out. This new understanding of the interdependency between adaptive sampling of visual information and photoreceptor structure explains readily why and how differently shaped photoreceptors of different fly species match the visual requirements of their lifestyle. A photoreceptor's maximum information transfer rate cannot be increased beyond its structural limits, hardwired in the maximum number microvilli and in their maximum bump production rate. Therefore, insect photoreceptors with the most and fastest microvilli in principle should provide the best vision. However, this comes at a cost: more microvilli also mean higher capacitance, and hence potentially slower membrane time constant, which can only be overcome by larger conductances, ultimately consuming more ATP [51, 52].

Supplemental References

1. Niven, J.E., Vahasoyrinki, M., Kauranen, M., Hardie, R.C., Juusola, M., and Weckstrom, M. (2003). The contribution of Shaker K+ channels to the information capacity of *Drosophila* photoreceptors. Nature *421*, 630-634.
2. Vähäsöyrinki, M., Niven, J.E., Hardie, R.C., Weckström, M., and Juusola, M. (2006). Robustness of neural coding in *Drosophila* photoreceptors in the absence of slow delayed rectifier K+ channels. J. Neurosci. *26*, 2652-2660.
3. Minke, B. (1977). Drosophila mutant with a transducer defect. Biophys Struct Mech *3*, 59-64.
4. Henderson, S.R., Reuss, H., and Hardie, R.C. (2000). Single photon responses in *Drosophila* photoreceptors and their regulation by Ca2+. J. Physiol. *524 Pt 1*, 179-194.
5. Juusola, M., and Hardie, R.C. (2001). Light adaptation in *Drosophila* photoreceptors: I. Response dynamics and signaling efficiency at 25 degrees C. J. Gen. Physiol. *117*, 3-25.
6. Faivre, O., and Juusola, M. (2008). Visual coding in locust photoreceptors. PLoS One *3*, e2173.
7. Liu, C.H., Satoh, A.K., Postma, M., Huang, J., Ready, D.F., and Hardie, R.C. (2008). Ca2+-dependent metarhodopsin inactivation mediated by calmodulin and NINAC myosin III. Neuron *59*, 778-789.
8. Cover, T., and Thomas, J. (1991). Elements of Information Theory, (New York: John Wiley & Sons Inc.).
9. Juusola, M., and de Polavieja, G.G. (2003). The rate of information transfer of naturalistic stimulation by graded potentials. J. Gen. Physiol. *122*, 191-206.
10. Franceschini, N., and Chagneux, R. (1997). Repetitive scanning in the fly compound eye. In Proceedings of the 25th Göttingen Neurobiology Conference 1997; Volume II. , N. Eisner and H. Wassle, eds. (Göttingen: Stuttgart, New York: Georg Thieme Verlag), p. 279.
11. Bahner, M., Frechter, S., Da Silva, N., Minke, B., Paulsen, R., and Huber, A. (2002). Light-regulated subcellular translocation of *Drosophila* TRPL channels induces long-term adaptation and modifies the light-induced current. Neuron *34*, 83-93.
12. Frechter, S., Elia, N., Tzarfaty, V., Selinger, Z., and Minke, B. (2007). Translocation of Gq alpha mediates long-term adaptation in *Drosophila* photoreceptors. J. Neurosci. *27*, 5571-5583.
13. Kiselev, A., Socolich, M., Vinos, J., Hardy, R.W., Zuker, C.S., and Ranganathan, R. (2000). A molecular pathway for light-dependent photoreceptor apoptosis in Drosophila. Neuron *28*, 139-152.
14. Kosloff, M., Elia, N., Joel-Almagor, T., Timberg, R., Zars, T.D., Hyde, D.R., Minke, B., and Selinger, Z. (2003). Regulation of light-dependent Gqalpha translocation and morphological changes in fly photoreceptors. Embo J. *22*, 459-468.
15. Pumir, A., Graves, J., Ranganathan, R., and Shraiman, B.I. (2008). Systems analysis of the single photon response in invertebrate photoreceptors. Proc. Natl. Acad. Sci. USA *105*, 10354-10359.
16. Huber, A., Sander, P., Gobert, A., Bahner, M., Hermann, R., and Paulsen, R. (1996). The transient receptor potential protein (Trp), a putative store-operated Ca2+ channel essential for phosphoinositide-mediated photoreception, forms a signaling complex with NorpA, InaC and InaD. Embo J. *15*, 7036-7045.
17. Hardie, R.C., and Raghu, P. (2001). Visual transduction in *Drosophila*. Nature *413*, 186-193.
18. Postma, M., Oberwinkler, J., and Stavenga, D.G. (1999). Does Ca2+ reach millimolar concentrations after single photon absorption in Drosophila photoreceptor microvilli? Biophys. J. *77*, 1811-1823.

19. Hofstee, C.A., Henderson, S., Hardie, R.C., and Stavenga, D.G. (1996). Differential effects of ninaC proteins (p132 and p174) on light-activated currents and pupil mechanism in Drosophila photoreceptors. Vis. Neurosci. *13*, 897-906.

20. Hardie, R.C. (1996). INDO-1 measurements of absolute resting and light-induced Ca2+ concentration in *Drosophila* photoreceptors. J. Neurosci. *16*, 2924-2933.

21. Porter, J.A., Yu, M., Doberstein, S.K., Pollard, T.D., and Montell, C. (1993). Dependence of calmodulin localization in the retina on the NINAC unconventional myosin. Science *262*, 1038-1042.

22. Maune, J.F., Klee, C.B., and Beckingham, K. (1992). Ca2+ binding and conformational change in two series of point mutations to the individual Ca(2+)-binding sites of calmodulin. J. Biol. Chem. *267*, 5286-5295.

23. Hardie, R.C., Martin, F., Cochrane, G.W., Juusola, M., Georgiev, P., and Raghu, P. (2002). Molecular basis of amplification in Drosophila phototransduction: roles for G protein, phospholipase C, and diacylglycerol kinase. Neuron *36*, 689-701.

24. Gonzalez-Bellido, P.T., Wardill, T.J., and Juusola, M. (2011). Compound eyes and retinal information processing in miniature dipteran species match their specific ecological demands. Proc. Natl. Acad. Sci. USA *108*, 4224-4229.

25. Juusola, M., and Hardie, R.C. (2001). Light adaptation in *Drosophila* photoreceptors: II. Rising temperature increases the bandwidth of reliable signaling. J. Gen. Physiol. *117*, 27-42.

26. Juusola, M., Kouvalainen, E., Järvilehto, M., and Weckström, M. (1994). Contrast gain, signal-to-noise ratio, and linearity in light-adapted blowfly photoreceptors. J. Gen. Physiol. *104*, 593-621.

27. Dodge, F.A., Knight, B.W., and Toyoda, J. (1968). Voltage Noise in Limulus Visual Cells. Science *160*, 88-&.

28. Wong, F., and Knight, B.W. (1980). Adapting-Bump Model for Eccentric Cells of Limulus. J. Gen. Physiol. *76*, 539-557.

29. Burton, B.G. (2006). Adaptation of single photon responses in photoreceptors of the housefly, Musca domestica: A novel spectral analysis. Vision Res. *46*, 622-635.

30. Wong, F., Knight, B.W., and Dodge, F.A. (1980). Dispersion of latencies in photoreceptors of *Limulus* and the adapting-bump model. J. Gen. Physiol. *76*, 517-537.

31. van Hateren, J.H. (1997). Processing of natural time series of intensities by the visual system of the blowfly. Vision Res. *37*, 3407-3416.

32. Suzuki, E., Katayama, E., and Hirosawa, K. (1993). Structure of photoreceptive membranes of Drosophila compound eyes as studied by quick-freezing electron microscopy. J. Electron Microsc. (Tokyo) *42*, 178-184.

33. Hochstrate, P., and Hamdorf, K. (1990). Microvillar Components of Light Adaptation in Blowflies. J. Gen. Physiol. *95*, 891-910.

34. Hardie, R.C., and Postma, M. (2008). Phototransduction in microvillar photoreceptors of *Drosophila* and other invertebrates. In The Senses: A Comprehensive Reference. Vision, Volume 1, A.I. Basbaum, A. Kaneko, G.M. Shepherd and G. Westheimer, eds. (San Diego: Academic Press), pp. 77-130.

35. Gillespie, D.T. (1976). General Method for Numerically Simulating Stochastic Time Evolution of Coupled Chemical-Reactions. J. Comput. Phys. *22*, 403-434.

36. Haseltine, E.L., and Rawlings, J.B. (2002). Approximate simulation of coupled fast and slow reactions for stochastic chemical kinetics. J. Chem. Phys. *117*, 6959-6969.

37. Puchalka, J., and Kierzek, A.M. (2004). Bridging the gap between stochastic and deterministic regimes in the kinetic simulations of the biochemical reaction networks. Biophys. J. *86*, 1357-1372.

38. Rao, C.V., and Arkin, A.P. (2003). Stochastic chemical kinetics and the quasi-steady-state assumption: Application to the Gillespie algorithm. J. Chem. Phys. *118*, 4999-5010.

39. Resat, H., Wiley, H.S., and Dixon, D.A. (2001). Probability-weighted dynamic Monte Carlo method for reaction kinetics simulations. J. Phys. Chem. B *105*, 11026-11034.

40. Salis, H., and Kaznessis, Y.N. (2005). An equation-free probabilistic steady-state approximation: Dynamic application to the stochastic simulation of biochemical reaction networks. J. Chem. Phys. *123*.

41. Luo, C.H., and Rudy, Y. (1994). A dynamic model of the cardiac ventricular action potential. I. Simulations of ionic currents and concentration changes. Circ. Res. *74*, 1071-1096.

42. Hardie, R.C. (1991). Voltage-sensitive potassium channels in *Drosophila* photoreceptors. J. Neurosci. *11*, 3079-3095.

43. Hardie, R.C., Voss, D., Pongs, O., and Laughlin, S.B. (1991). Novel potassium channels encoded by the Shaker locus in *Drosophila* photoreceptors. Neuron *6*, 477-486.

44. Krause, Y., Krause, S., Huang, J., Liu, C.H., Hardie, R.C., and Weckstrom, M. (2008). Light-dependent modulation of Shab channels via phosphoinositide depletion in *Drosophila* photoreceptors. Neuron *59*, 596-607.

45. Weckström, M., Hardie, R.C., and Laughlin, S.B. (1991). Voltage-activated potassium channels in blowfly photoreceptors and their role in light adaptation. J. Physiol. *440*, 635-657.

46. Laughlin, S.B., and Weckstrom, M. (1993). Fast and Slow Photoreceptors - a Comparative-Study of the Functional Diversity of Coding and Conductances in the Diptera. J. Comp. Physiol. A *172*, 593-609.

47. Juusola, M., and Weckstrom, M. (1993). Band-pass filtering by voltage-dependent membrane in an insect photoreceptor. Neurosci. Lett. *154*, 84-88.

48. Shannon, C.E. (1948). A Mathematical Theory of Communication. Bell Syst. Tech. J. *27*, 379-423.

49. Zheng, L., de Polavieja, G.G., Wolfram, V., Asyali, M.H., Hardie, R.C., and Juusola, M. (2006). Feedback network controls photoreceptor output at the layer of first visual synapses in *Drosophila*. J. Gen. Physiol. *127*, 495-510.

50. Zheng, L., Nikolaev, A., Wardill, T.J., O'Kane, C.J., de Polavieja, G.G., and Juusola, M. (2009). Network adaptation improves temporal representation of naturalistic stimuli in *Drosophila* eye: I dynamics. PLoS One *4*, e4307.

51. Fain, G.L., Hardie, R., and Laughlin, S.B. (2010). Phototransduction and the evolution of photoreceptors. Curr. Biol. *20*, R114-124.

52. Laughlin, S.B., de Ruyter van Steveninck, R.R., and Anderson, J.C. (1998). The metabolic cost of neural information. Nat. Neurosci. *1*, 36-41.
